# The scaffold nucleoporins SAR1 and SAR3 are essential for proper meiotic progression in *Arabidopsis thaliana*

**DOI:** 10.3389/fcell.2023.1285695

**Published:** 2023-12-04

**Authors:** Nadia Fernández-Jiménez, Marina Martinez-Garcia, Javier Varas, Félix Gil-Dones, Juan Luis Santos, Mónica Pradillo

**Affiliations:** ^1^ Department of Genetics, Physiology and Microbiology, Faculty of Biological Sciences, Universidad Complutense de Madrid, Madrid, Spain; ^2^ Department of Biotechnology-Plant Biology, School of Agricultural, Food and Biosystems Engineering, Universidad Politécnica de Madrid, Madrid, Spain; ^3^ GlaxoSmithKline Spain, Madrid, Spain

**Keywords:** Arabidopsis, meiosis, nuclear pore complex, NUP96, NUP160, SAR1, SAR3

## Abstract

Nuclear Pore Complexes (NPCs) are embedded in the nuclear envelope (NE), regulating macromolecule transport and physically interacting with chromatin. The NE undergoes dramatic breakdown and reformation during plant cell division. In addition, this structure has a specific meiotic function, anchoring and positioning telomeres to facilitate the pairing of homologous chromosomes. To elucidate a possible function of the structural components of the NPCs in meiosis, we have characterized several Arabidopsis lines with mutations in genes encoding nucleoporins belonging to the outer ring complex. Plants defective for either SUPPRESSOR OF AUXIN RESISTANCE1 (SAR1, also called NUP160) or SAR3 (NUP96) present condensation abnormalities and SPO11-dependent chromosome fragmentation in a fraction of meiocytes, which is increased in the double mutant *sar1 sar3*. We also observed these meiotic defects in mutants deficient in the outer ring complex protein HOS1, but not in mutants affected in other components of this complex. Furthermore, our findings may suggest defects in the structure of NPCs in *sar1* and a potential link between the meiotic role of this nucleoporin and a component of the RUBylation pathway. These results provide the first insights in plants into the role of nucleoporins in meiotic chromosome behavior.

## Introduction

The nuclear pore complex (NPC) is one of the largest non-polymeric protein complexes in eukaryotic cells (Knockenhauer and Schwartz, 2016). The main function of the NPC is to mediate the selective nucleocytoplasmic transport of macromolecules while allowing the free diffusion of molecules smaller than 40 kDa (Raíces and D’Angelo, 2012; Meier et al., 2017). Most of the proteins forming the NPCs, known as nucleoporins, are evolutionarily conserved, as well as the octagonal symmetry of these complexes (Gall, 1967; Hoelz et al., 2011; Lin and Hoelz, 2019). The NPC is organized in different subcomplexes composed of more than 30 different nucleoporins (Tamura and Hara-Nishimura, 2013; Li and Gu, 2020). In addition to their main role as trafficking channels, NPCs also act as hubs for relevant processes such as chromatin organization, gene transcription, replication stress resolution or DNA repair (Blobel, 1985; Beck and Hurt, 2017; Lamm et al., 2021). In these cases, NPCs could act as membrane-bound sliding platforms to associate the underlying chromatin with other protein complexes localized in the nucleus (Strambio-De-Castilla et al., 2010; Raices and D’Angelo, 2012).

The overall organization of the NPCs is highly conserved among evolutionarily distant eukaryotes, although there is a significant variability in the composition of nucleoporins (Fiserova et al., 2009; Tamura et al., 2011; 2013; Fernandez-Martinez and Rout, 2021). Nucleoporins assemble into different subcomplexes forming the inner, outer, and membrane rings. Moreover, the central channel is filled by phenylalanine-glycine-rich (FG) nucleoporins, and a nuclear basket and cytoplasmic filaments are anchored to the nuclear and cytoplasmic outer rings, respectively (Alber et al., 2007; Beck and Hurt, 2017; Meier et al., 2017). The outer ring complex nucleoporins form Y-shaped complexes, and accordingly, this NPC module is also known as the Y-complex (Stuwe et al., 2015). This complex is also called NUP107-160 in plants and vertebrates and Nup84 (or Nup84-Nup133) in yeast (Lutzmann et al., 2002; Walther et al., 2003; Meier et al., 2017; Nordeen et al., 2020). The outer ring complex plays essential roles in NPC assembly, microtubule polymerization at kinetochores, and DNA repair (Walther et al., 2003; Nagai et al., 2008; Mishra et al., 2010). In *Arabidopsis thaliana*, members of this complex are involved in flowering time regulation, abiotic stress and immune responses, as well as in hormone signaling (Gu, 2018; Zhang A. et al., 2020; Cheng et al., 2020; Nie et al., 2023). In this species, defective mutants for members of this complex, such as *nup160* and *nup96*, were identified in a screening for suppression of auxin resistance phenotypes. For this reason, these mutants are also called *suppressor of auxin resistance1* (*sar1*) and *sar3*, respectively, and exhibit pleiotropic growth defects including an early flowering phenotype (Cernac et al., 1997; Parry et al., 2006; Wiermer et al., 2012).

Meiosis is a specialized cell division required to generate haploid gametes from diploid parent cells. During early prophase I, homologous chromosomes pair, synapse and exchange genetic information. These processes are facilitated by the movement of telomeres along the nuclear envelope (NE) (Koszul and Kleckner, 2009). The transmission of cytoplasmic forces to telomeres is mediated by the LINC (LInker of the Nucleoskeleton and Cytoskeleton) complexes (Starr, 2009; Klutstein and Cooper, 2014). These complexes consist of SUN and KASH proteins that span the inner and outer nuclear membranes (INM and ONM), connecting nuclear and cytoplasmic structures. Disrupting the function of the LINC complex impairs chromosome movements, leading to defects in synapsis and meiotic recombination (Ding et al., 2007; Murphy et al., 2014; Varas et al., 2015; Zhang F. et al., 2020). This role for LINC complexes seems to be conserved in yeast, animals, and plants (Burke, 2018). It has been suggested that LINC complexes and NPCs could be functionally related. Indeed, in HeLa cells SUN1 interacts with NPCs being important for their distribution (Liu et al., 2007). However, studies showing a possible meiotic function for NPCs are scarce and mostly focused on yeast. Several of these studies have associated the function of certain nucleoporins with kinetochores and chromosome segregation (Yang et al., 2015; Hattersley et al., 2016; 2022). In *Saccharomyces cerevisiae*, the nuclear basket nucleoporins Nup2 and Nup60 transiently detach from the NPC core during the first meiotic division and promote chromosome dynamics during meiosis (Chu et al., 2017; Komachi and Burgess, 2022; King et al., 2023). Until now, no work in plants has linked NPCs to chromatin organization and chromosome behavior during meiosis. In this regard, it is important to note that in contrast to the situation in yeast, in plants, the NE breaks down to allow the connection between the chromosomes and the cytoplasmic spindle (Meier et al., 2017).

In this study, we focus on the meiotic role of nucleoporins belonging to the outer ring complex of the NPC, in particular NUP160 (SAR1) and NUP96 (SAR3). Analysis of the meiotic process in pollen mother cells (PMCs) has revealed that SAR1 and SAR3 are essential for ensuring proper chromatin condensation and meiotic repair of double-strand breaks (DSBs). Additionally, the findings may suggest that SAR1 is important for preserving the integrity of NPCs in prophase I and that its meiotic function could be linked to that of AXR1, a subunit of the RUB1 activating enzyme, which regulates the protein degradation activity of SKP1-CULLIN1-F-BOX (SCF) complexes. Our work provides, for the first time, important insights into the function of NPCs in plant meiosis.

## Materials and methods

### Plant material and growth conditions

All plants used in this study were *Arabidopsis thaliana* (L.) Heynh, Columbia (Col-0) accession. Mutant lines were obtained from the European Arabidopsis Stock Centre: *sar1-4* (SALK_126801), *sar3-4* (SALK_117966), *hos1-3* (SALK_069312), *nup85-2* (SALK_113274), and *seh1-1* (SALK_022717). Double mutants were built using: *spo11-1-5* (SALK_009440) (Pradillo et al., 2007), and *axr1-31* (SALK_013238) (Martínez-García et al., 2020). Seeds were sown on soil directly or after transfer from MS plates (Murashige and Skoog, 1962). Plants were grown under long-day conditions (16 h light/8 h dark) at 19°C. Homozygous plants were identified by PCR screening using primers listed in Supplementary Table S1.

### Cytogenetic analyses

Fixation of flower buds and spreading of male meiocytes were performed according to Ross et al. (1996). The fluorescent *in situ* hybridization (FISH) technique was carried out as described by Sanchez-Moran et al. (2001) with minor modifications. The DNA probes used in the analysis of chromosomal configurations at metaphase I were 45S rDNA (pTa71; Gerlach and Bedbrook, 1979) and 5S rDNA (pCT4.2; Campell et al., 1992). At least three plants of each genotype were analyzed. To analyze pollen grains, we used fresh material and transferred anthers to a 1% solution of carmine in 45% acetic acid, we heated the slide slowly over an alcohol burner (∼30 s) and used the squash method. A Nikon Eclipse E400 phase-contrast microscope with a Nikon DMX-12005-E400 digital camera were used for image acquisition.

To detect meiotic recombination proteins, we performed the spreading technique described by Armstrong et al. (2009) with the modifications included in Varas and Pradillo (2018). To detect histone modifications and NE proteins, we applied the squash technique detailed in Oliver et al. (2013). Information about the dilution and source of primary antibodies is included in Supplementary Table S2. The secondary antibodies used were anti-rabbit Alexa Fluor 555-conjugated (Invitrogen, Molecular probes; 1:500), anti-rat Alexa Fluor 555-conjugated (Invitrogen, Molecular probes; 1:500), anti-mouse FITC-conjugated (Agrisera; 1:100), and anti-rabbit FITC-conjugated (Merck; 1:50). Cells were imaged using an Olympus BX61 epifluorescence microscope with an Olympus DP71 digital camera. Quantification of foci was performed using ImageJ. We scored all images blind to genotype.

### RNA FISH

To evaluate mRNA nuclear accumulation, we followed the protocol described in Parry (2014) using samples from roots and flower buds. The samples were incubated with a Cy3 labelled oligo(dT) probe. For quantification, we compared pixel intensity between the nucleus and the cytoplasm (ImageJ) and calculated the fold change (ratio nucleus/cytoplasm) in each cell respect to Col-0. Cells from at least three different slides were analyzed for each tissue.

### Statistical analyses

Microsoft Office Excel and GraphPad Prism were used for data organization and statistical analysis, respectively. Mann-Whitney *U* test and one-way ANOVA with *post hoc* Tukey test were performed to compare independent samples. Fisher’s Exact test and Chi-square test were used to compare frequencies.

## Results

### Mutants defective in outer ring complex nucleoporins show abnormal male meiosis

The mutants analyzed in this study were previously characterized by Cernac et al. (1997) and Parry et al. (2006). These authors revealed that *sar1* and *sar3* show a pleiotropic phenotype with early flowering, dwarfism, and abnormal auxin response. These mutants also have altered expression of certain defense genes and nuclear mRNA accumulation (Meier and Brkljacic, 2009; Wiermer et al., 2012). In addition, the pleiotropic defects in the single mutants were exacerbated in the double mutant *sar1 sar3*, suggesting the disruption of an NPC specific function associated to the loss of several subunits (Parry et al., 2006). The reduced production of seeds in these plants was attributed to their developmental defects and abnormal inflorescences (Parry et al., 2006).

**FIGURE 1:**
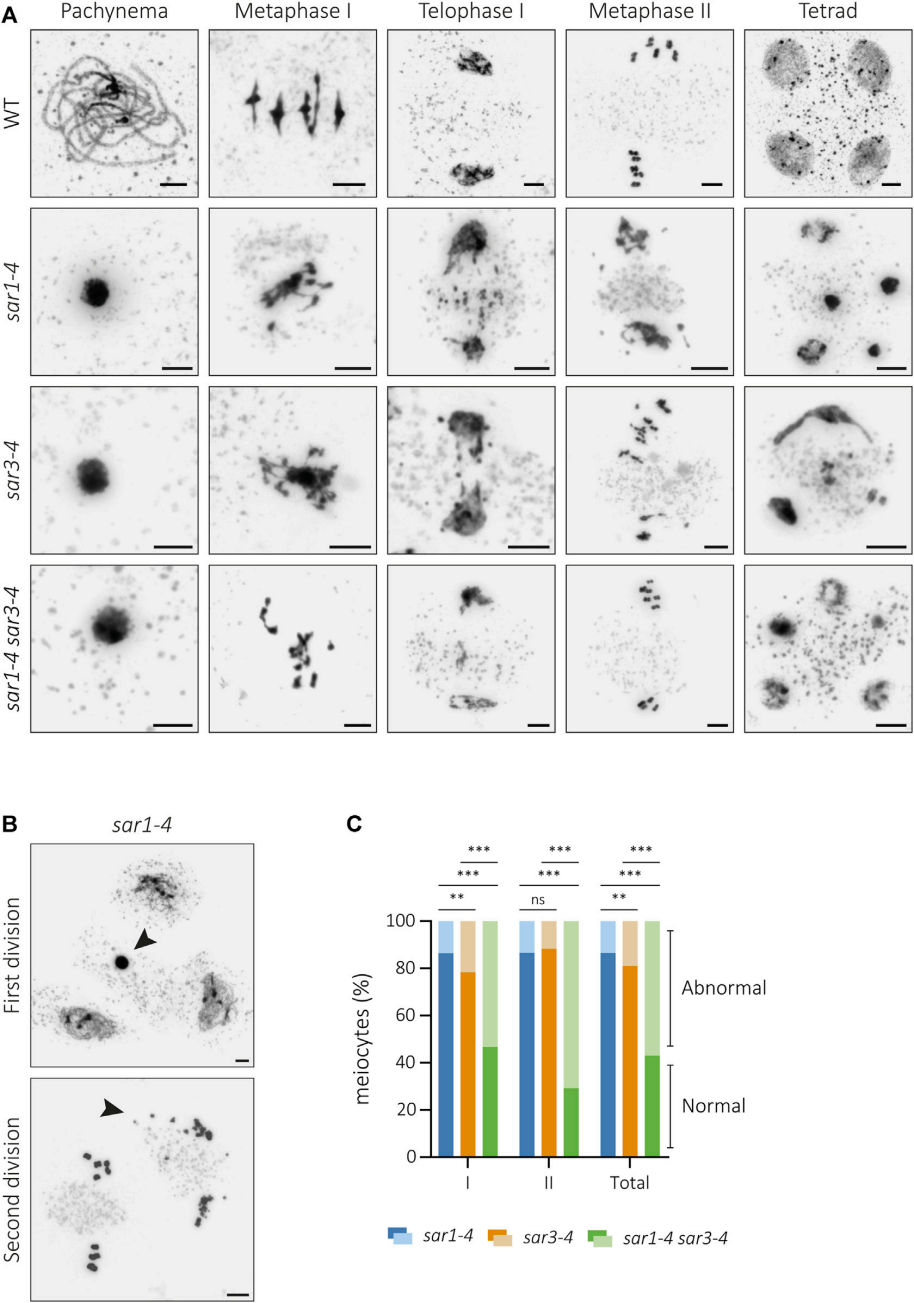
Cytological analysis of meiotic defects in *sar* mutants. **(A)** Chromosome spreads of male meiocytes from WT, *sar1-4, sar3-4* and the double mutant *sar1-4 sar3-4.* Mutants show hypercondensed meiocytes at pachynema, entangled chromosomes at metaphase I, chromosome fragments and unbalanced segregations at telophase I, unbalanced nuclei and chromosome fragments at metaphase II, and polyads with nuclei displaying unevenly condensed chromatin. **(B)** Examples from *sar1-4* showing abnormal meiocytes (arrowheads) surrounded by normal meiocytes in both meiotic divisions. **(C)** Proportion of abnormal and normal meiocytes in *sar1-4, sar3-4* and the double mutant *sar1-4 sar3-4*. Fisher’s exact test was performed to analyze differences between mutants (*p*-value: ns–non-significant, *p < 0.05, **p < 0.01, ***p < 0.001). I: Meiosis I; II: Meiosis II. Scale bars = 5 µm.

In this work, we have confirmed the fertility defects in *sar1* and *sar3* (Supplementary Figure S1) and observed that this phenotype is aggravated in the double mutant *sar1 sar3*, which is completely sterile. In addition, this double mutant has viability problems, as only 2% of *sar1 sar3* double mutant plants were obtained in the offspring of double heterozygous plants. The detailed cytological characterization of pollen mother cells (PMCs) that we conducted in these mutants revealed that these fertility defects are due to abnormalities during meiosis. In the single mutants *sar1* and *sar3*, most of the meiocytes were apparently indistinguishable from the control: homologous chromosomes were associated along their entire length in pachynema; five bivalents, with no alterations in chromosome condensation, were observed at metaphase I; and chromosomes and sister-chromatids segregated correctly at anaphase I and II, respectively, giving rise to balanced tetrads with the same amount of genetic material in each nucleus (Supplementary Figure S2). However, alterations in chromatin condensation and chromosome fragmentation appeared at different stages corresponding to both meiotic divisions in a percentage of meiocytes (*sar1*: 13.52%, n = 636; *sar3*: 19.08%, n = 1,373). No anomalies were observed at leptonema, but from zygonema onwards some PMCs showed aberrant chromatin condensation. In metaphase I, entangled bivalents were observed and segregation problems, as well as chromosome fragments, were detected in both meiosis I and II. All these defects led to the formation of tetrads and polyads with differentially condensed and unbalanced nuclei (Figure 1A). Abnormal meiocytes were not limited to specific flower buds or anthers but appeared alongside populations of normal cells (Figure 1B; Supplementary Figure S1). The percentage of abnormal meiocytes was much higher in the double mutant (57.02%, n = 114) than in the single mutants, which also differed from each other (Figure 1C; Supplementary Tables S3, S4). In addition, the proportion of meiocytes with chromosomal fragmentation was increased in the double mutant compared to the single mutants (Supplementary Table S5).

In order to elucidate whether these defects were a consequence of the alteration of any component of the outer ring complex, we analyzed other mutants defective for this subunit. HIGH EXPRESSION OF OSMOTICALLY RESPONSIVE GENES1 (HOS1) functions in the regulation of flowering through controlling the protein level of CONSTANT, like SAR1 and SAR3 (Cheng et al., 2020; Li et al., 2020). The cytological analysis of male meiosis in *hos1* revealed the same type of alterations as those observed in *sar1* and *sar3* mutants (Supplementary Figure S2). Problems in chromatin condensation and chromosomal fragmentation were detected, although meiocytes without any abnormalities could also be observed. Interestingly, these meiotic alterations do not appear when other components of the outer ring, such as NUP85 (n = 166) or SEH1 (n = 36), are absent (Supplementary Figure S2). Both *sar1* (mutant in which we detected meiosis abnormalities) and *nup85* (mutant in which we did not detect any meiosis abnormalities) show mRNA accumulation in the nucleus, not only in root cells, but also in flower bud cells (Supplementary Figure S3). Therefore, these results show that the different outer ring components are not equally important during meiosis, and that the meiotic defects do not appear to arise as a consequence of the mRNA accumulation.

### Synapsis and chiasma frequency, as well as the pattern of certain epigenetic marks, are normal in most *sar1* meiocytes

**FIGURE 2:**
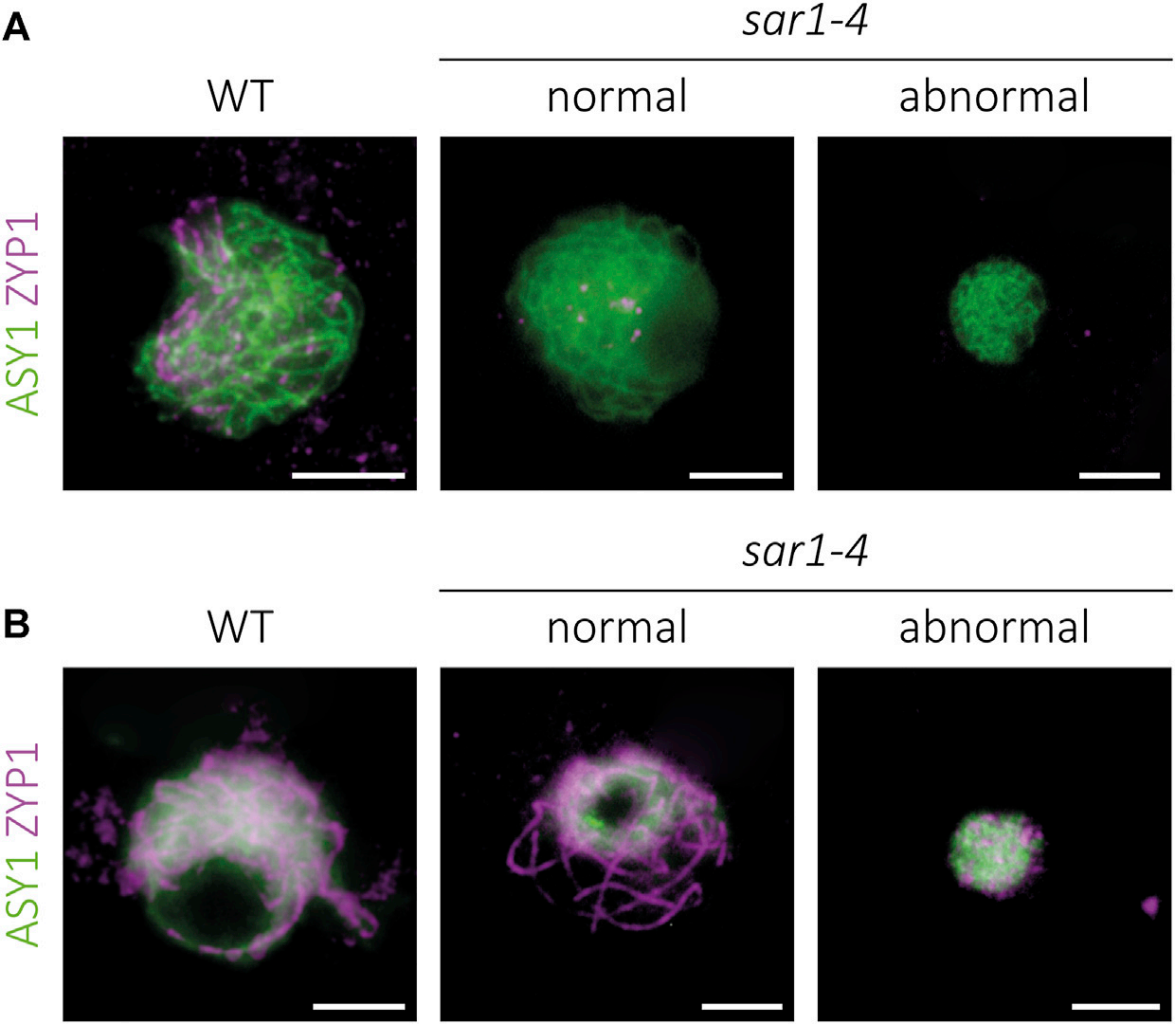
Immunolocalization of meiotic chromosome axes and synaptonemal complex in *sar1-4*. Squash preparations of male meiocytes showing the meiotic chromosome axis protein ASY1 (green) and the synaptonemal complex transverse filament protein ZYP1 (magenta). **(A)** Zygonema. Chromosome axes appear to be normal despite condensation defects in *sar1-4*. **(B)** Pachynema. Although full synapsis is observed in normal-looking *sar1-4* meiocytes, ZYP1 forms numerous chromosomic aberrant aggregates in hypercondensed *sar1-4* meiocytes, revealing problems in synaptonemal complex formation in these cells. Scale bars = 5 µm.

In order to conduct a more exhaustive study of the meiotic process, the *sar1* mutant was chosen as representative of the meiotic problems observed in the outer ring complex mutants. Since the meiotic problems began to be detected in zygonema-pachynema, we carried out an analysis of the synaptonemal complex (SC) formation by immunolocalization of ASY1 (protein associated to the axial/lateral element) and ZYP1 (transverse filament protein) on prophase I chromosome spreads (Armstrong et al., 2002; Higgins et al., 2005). We also detected two cell populations with respect to SC formation. In all meiocytes in which the chromosome morphology was indistinguishable to the WT (Supplementary Figure S2), the pattern corresponding to the ASY1+ZYP1 proteins revealed normal behavior: bright linear signals of ASY1 in the unsynapsed regions during zygonema and short stretches of ZYP1 that extended until full synapsis at pachynema (Figure 2; Supplementary Figure S4). In the case of meiocytes with extreme chromosomal condensation, we observed a continuous ASY1 signal in zygonema, despite the small size of these nuclei (Figure 2A). However, in these abnormal meiocytes we could not detect a continuous ZYP1 signal, observing only some aggregates without a defined pattern (Figure 2B). Thus, chromosomal axes appear to form correctly in *sar1* meiocytes, and although synapsis is normal in most *sar1* meiocytes, some have problems achieving synapsis.

**FIGURE 3:**
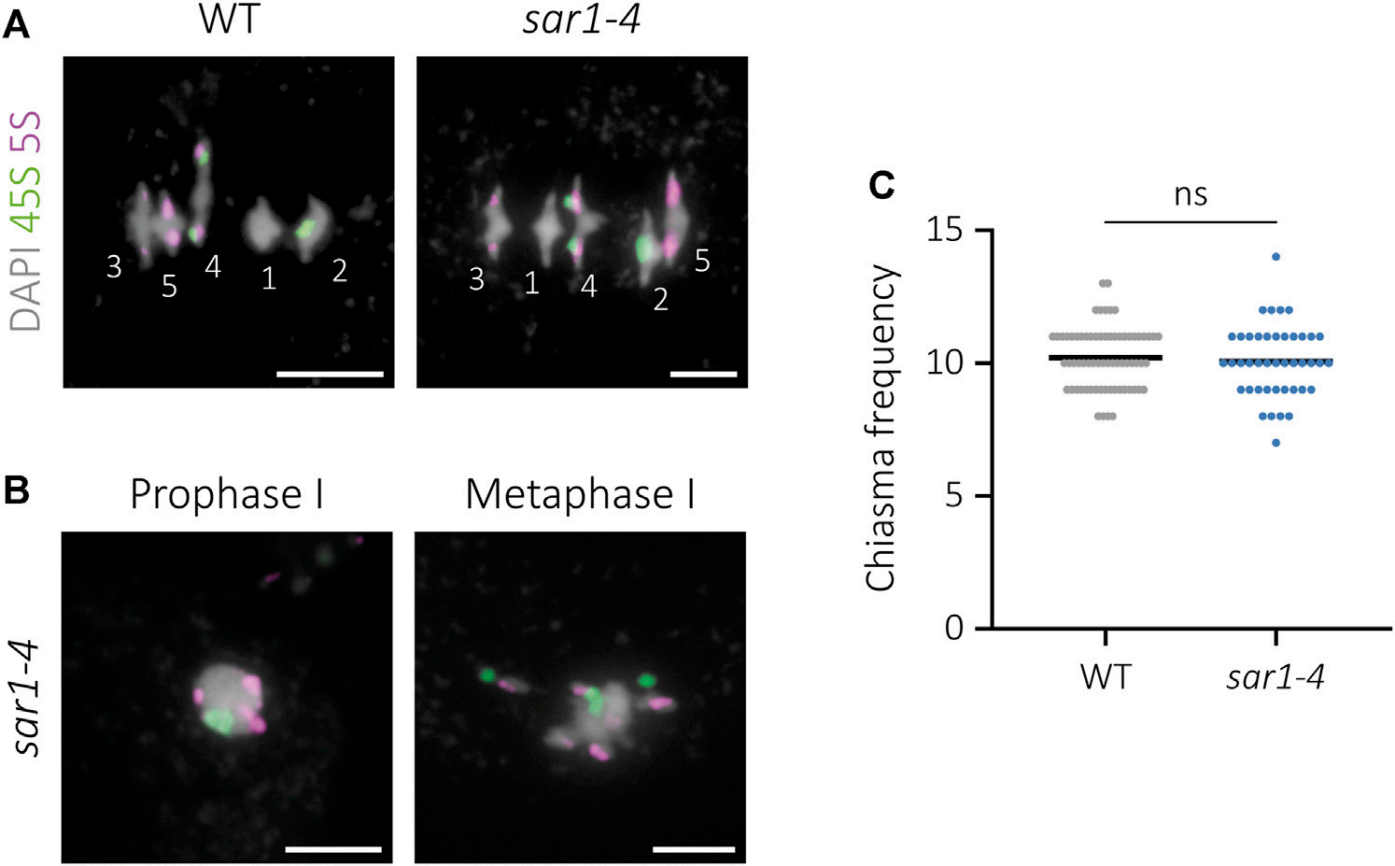
Cytological analysis of chiasma frequency in *sar1-4*. 45S rDNA (green) and 5S rDNA (magenta) probes were used for chromosome identification. DAPI is showed in gray. **(A)** WT and normal-looking *sar1-4* meiocytes at metaphase I. Numbers identify each bivalent. **(B)** Abnormal *sar1-4* meiocytes. The position of the FISH signals indicates pairing in prophase I and some co-orientation of homologous chromosomes in metaphase I. **(C)** Quantification of chiasma frequency per cell in WT and *sar1-4* (see the text for more details). Scale bars = 5 µm.

To determine whether, despite not detecting problems in synapsis and bivalent formation, there is any defect in meiotic recombination in normal-looking meiocytes in *sar1*, the frequency of chiasmata per cell at metaphase I was analyzed. To facilitate the interpretation of bivalent morphology and the localization of chiasmata, we performed 45S and 5S rDNA FISH (Sanchez-Moran et al., 2001) (Figure 3A). This analysis could not be applied to meiocytes with aberrant chromatin condensation, although we observed an arrangement of the FISH signals indicating some level of pairing, since homologous chromosomes were close together in the nucleus and presented some co-orientation at metaphase I (Figure 3B). The mean cell chiasma frequency in the WT was 10.20 ± 0.14 (n = 69), with a range of variation from 8 to 13. In *sar1* no significant differences were found with respect to this value, since the mean was 10.07 ± 0.21 (n = 43), varying from 7 to 14 (*U* = 1,393; *p* = 0.579) (Figure 3C).

Histone post-translational modifications are thought to play a pivotal role in chromosome condensation during meiosis (Fuchs et al., 2006; Xu et al., 2009). To test whether there was any variation in the epigenetic pattern of abnormally condensed meiocytes, we immunolocalized histone modifications associated to euchromatin and heterochromatin, as well as a modification specific of the chromosomal condensation process. Specifically, we analyzed the pattern corresponding to H3K4me3 (euchromatin-specific methylation), H3K9me2 (heterochromatin-specific methylation), and H3S10ph (phosphorylation associated to chromosome condensation) (Supplementary Figure S5). H3K4me3 is observed in all chromosomal regions except pericentromeric heterochromatin (Oliver et al., 2013). No variations from the WT were detected in *sar1* prophase I meiocytes. In the case of *sar1* hypercondensed meiocytes, H3K4me3 also had a similar pattern, appearing in most of the chromatin area. On the other hand, H3K9me2, which is restricted to pericentromeric regions throughout meiosis (Oliver et al., 2013), showed no variations in *sar1* meiocytes compared to WT, since signals were always observed in the chromocenters or brightest DAPI regions. Surprisingly, no changes were observed in the H3S10ph pattern either. This mark appears in Arabidopsis from diplonema onwards, a stage in which the chromatin is more condensed (Oliver et al., 2013). We could expect the presence of this modification in *sar1* meiocytes with hypercondensation. However, no signal corresponding to this epigenetic mark was detected despite chromatin compaction. Therefore, the condensation abnormalities observed in *sar1* meiocytes are not due to alterations in these histone modifications, at least at the cytological level.

### Chromosomal fragmentation defects observed in *sar1* are SPO11-dependent

Analysis of PMCs from *sar1* plants revealed fragmented chromosomes from anaphase I onwards in a percentage of meiocytes, leading to the formation of polyads containing microspores with unequal amounts of DNA (Figure 1). To ascertain whether DSBs formed by SPO11 could be the source of the chromosome fragmentation observed in SAR1-deficient plants, we generated *sar1 spo11-1* double mutants. Meiosis in *spo11* mutants is characterized by the presence of ten univalents at metaphase I, which segregate randomly during anaphase I (Grelon et al., 2001). In the absence of either SPO11-1 or SPO11-2, no DSBs occur at the onset of meiosis, therefore the integrity of the chromosomes in *spo11* mutants is intact.

**FIGURE 4:**
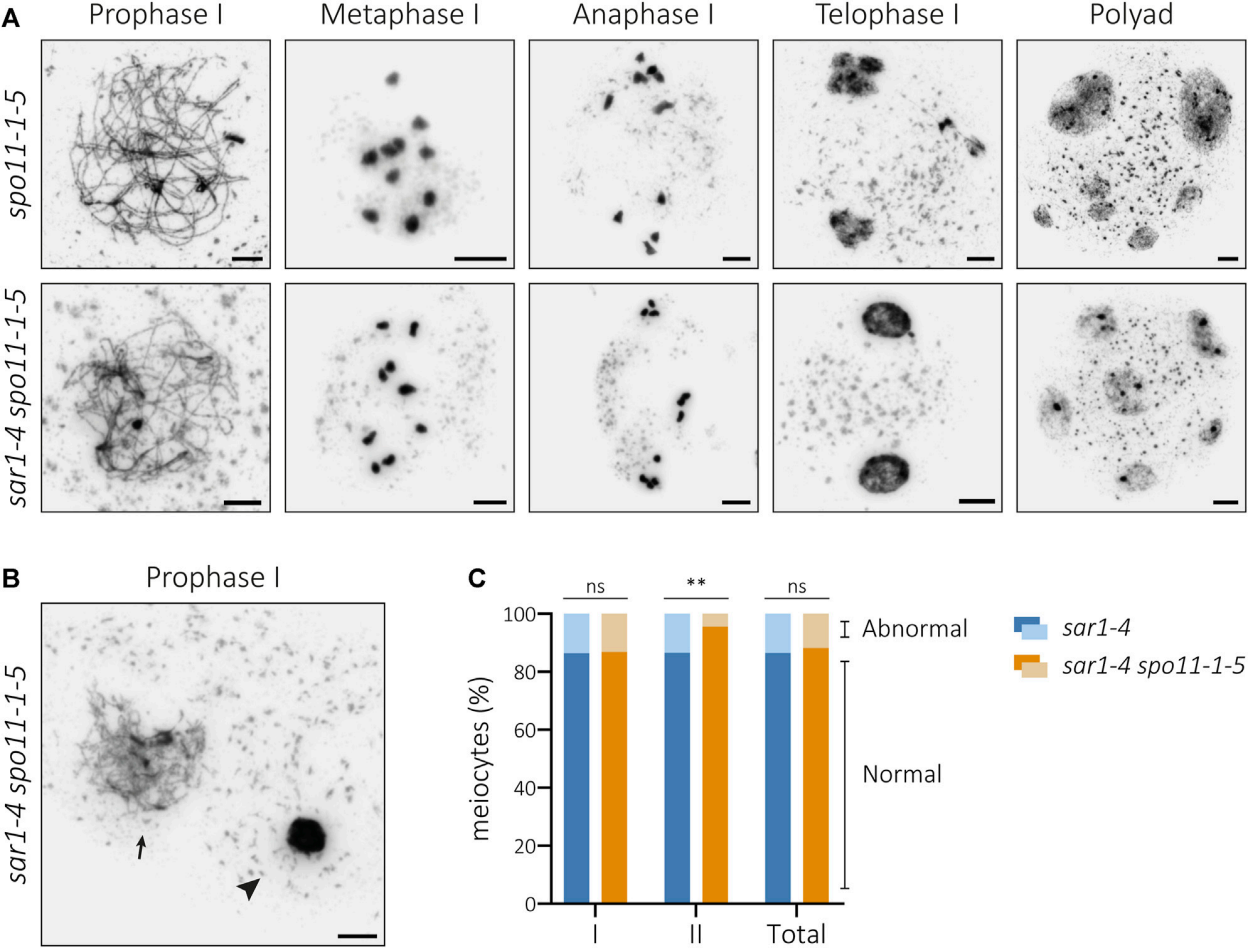
Cytological analysis of PMCs from the double mutant *sar1-4 spo11-1-5*. **(A)** Chromosome spreads of male meiocytes in *spo11-1-5* and *sar1-4 spo11-1-5*. Homologous chromosomes fail to undergo synapsis and ten univalents are observed at metaphase I in both mutants. The presence of univalents leads to mis-segregations of chromosomes at anaphase I, unbalanced nuclei during second meiotic division and polyads. **(B)** Example of hypercondensed (arrowhead) and normal-looking (arrow) meiocyte in the double mutant *sar1-4 spo11-1-5*. **(C)** Proportion of abnormal and normal meiocytes in *sar1-4* (blue) and *sar1-4 spo11-1-5* (orange). Fisher’s exact test was performed to analyze differences between the mutants (*p*-value: ns–non-significant, *p < 0.05, **p < 0.01, ***p < 0.001). I: Meiosis I; II: Meiosis II. Scale bars = 5 µm.

The meiotic phenotype of the double mutant *sar1 spo11-1* was very similar to that observed in the *spo11-1* single mutant, and no formation of SC or bivalents was detected. Ten univalents were invariably observed at metaphase I (n = 42) and no evidence of chromosomal fragmentation was found in any of the successive stages of meiosis (Figure 4A). Therefore, *sar1* chromosomal fragmentation problems are caused by the failure to repair SPO11-induced DSBs.

Detailed analysis of *sar1 spo11-1* PMCs revealed that although chromosomal fragmentation disappears, this double mutant still shows alterations in chromatin condensation (Figure 4B). In fact, the percentage of abnormal meiocytes (with alterations in chromatin condensation and/or chromosome fragmentation) during the first division did not vary significantly from that observed in the *sar1* single mutant (Figure 4C; Supplementary Table S6). In contrast, we detected a reduction in the frequency of abnormal meiocytes observed during the second division in the double mutant. The differences become apparent at the second division because at this division most of the abnormal meiocytes quantified in the *sar1* single mutant have chromosomal breaks, whereas at the first division most of the abnormal *sar1* meiocytes have chromatin condensation problems. Therefore, the chromosome condensation abnormalities observed in the *sar1* single mutant do not appear to arise from a specific meiotic alteration or at least from defects in the processing of DSBs. There is no evidence to suggest that hypercondensation has a pre-meiotic nature, as we did not identify any issues with chromatin condensation in somatic cells (Supplementary Figure S5). Furthermore, as mentioned before, all the observed leptotene meiocytes appeared to be normal-looking.

### γH2AX and RAD51 foci are significantly reduced in hypercondensed *sar1* meiocytes

**FIGURE 5:**
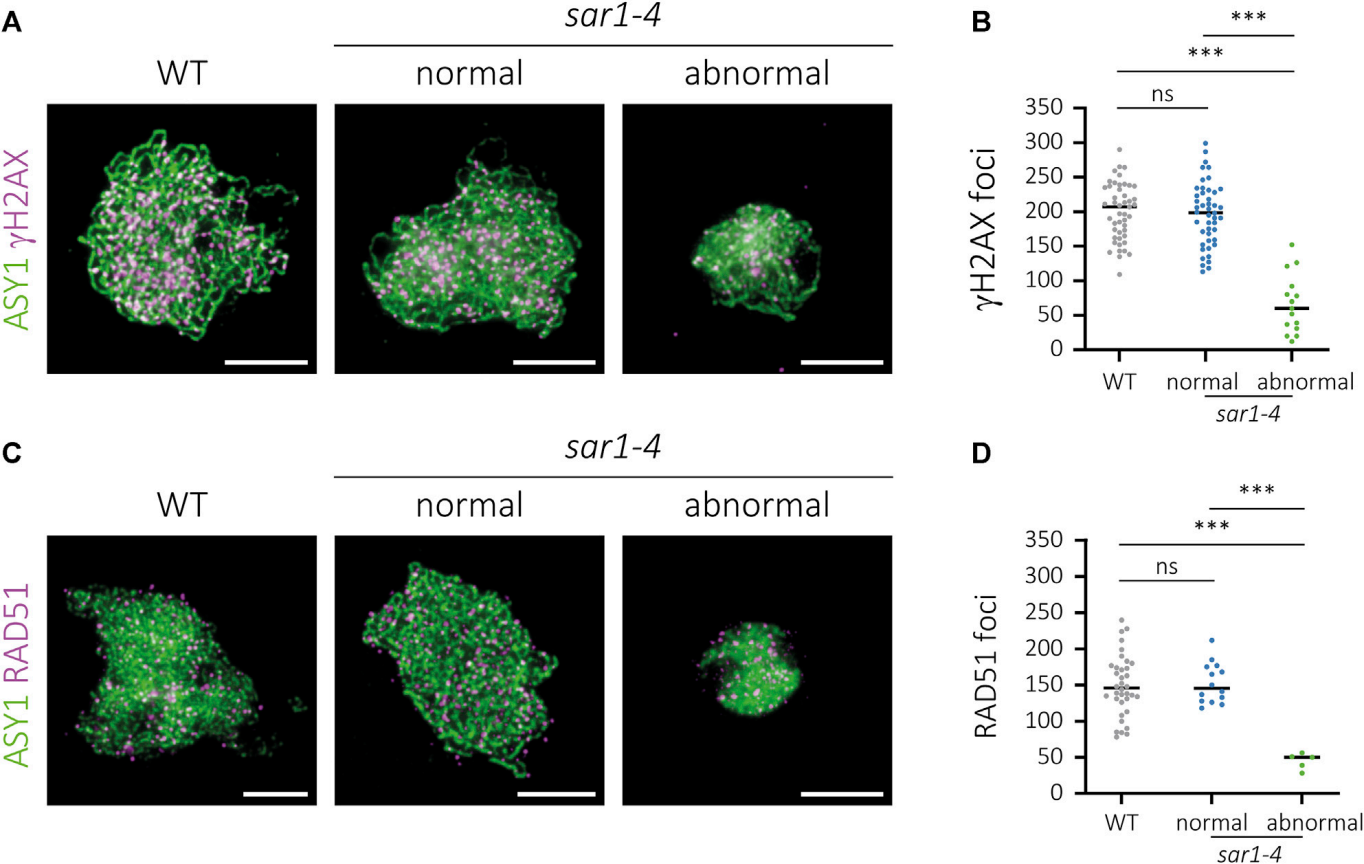
Immunolocalization of γH2AX and RAD51 in *sar1-4*. Chromosome spreads of male meiocytes showing γH2AX (magenta) and the recombinase RAD51 (magenta). ASY1 (green) has been used as a cytological marker to identify the meiotic chromosome axes. **(A)** WT, normal-looking and hypercondensed *sar1-4* zygotene cells showing ASY1 (green) and γH2AX foci (magenta). **(B)** Quantification of γH2AX foci in zygotene cells. **(C)** WT, normal-looking and hypercondensed *sar1-4* zygotene cells showing ASY1 (green) and RAD51 foci (magenta). **(D)** Quantification of RAD51 foci in zygotene cells. One-way ANOVA with a Tukey’s *post hoc* test was performed in both cases (*p*-value: ns–non-significant, *p* *** < 0.001). Scale bars = 5 µm.

To further examine the meiotic homologous recombination (HR) process in *sar1* meiocytes, we detected phosphorylated histone H2AX (γH2AX) and RAD51 foci by immunolocalization. γH2AX is deposited at DNA damage sites and is commonly used as a DSB-marker (Lowndes and Toh, 2005), and the recombinase RAD51 is loaded on ssDNA during meiotic recombination (Kurzbauer et al., 2012). The number of foci corresponding to both proteins was quantified in both normal appearing and hypercondensed *sar1* meiocytes (Figure 5; Supplementary Figure S6). We used ASY1 protein as a prophase I progression marker (Armstrong et al., 2002).

The number of γH2AX foci in *sar1* normal-looking meiocytes (196.61 ± 6.76; n = 46) was comparable as that observed in WT meiocytes (200.04 ± 6.02; n = 47), whereas we detected a significant reduction in the number of γH2AX foci in *sar1* hypercondensed meiocytes (66,00 ± 10,96; n = 15) (Figures 5A, B; Supplementary Table S7). The results for the quantification of the number of RAD51 foci were very similar, as no differences were found between normal-looking *sar1* (152.71 ± 7.49; n = 14) and WT meiocytes (149.42 ± 7.03; n = 36), whereas the number of RAD51 foci was drastically reduced in *sar1* hypercondensed cells (44.8 ± 5.03; n = 5) (Figures 5C, D; Supplementary Table S7). These results confirm that the meiotic recombination process, in line with the results obtained for synapsis, is severely compromised in *sar1* hypercondensed meiocytes.

### Nuclear envelope distribution of NPCs is altered in abnormal *sar1* meiocytes

**FIGURE 6:**
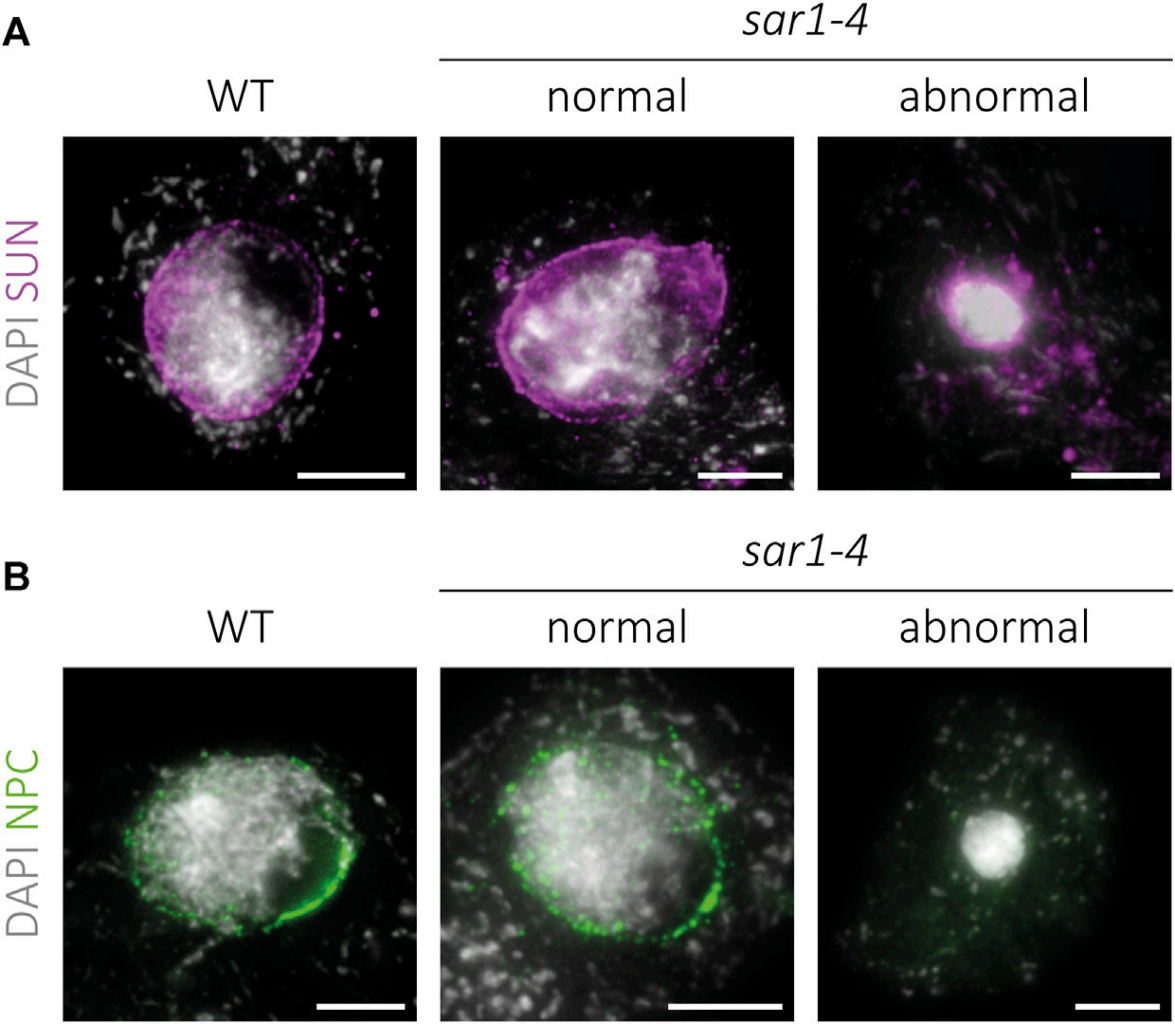
Immunolocalization of SUN proteins and NPCs in *sar1-4*. Squash preparations of WT, normal-looking and hypercondensed *sar1-4* zygotene cells. **(A)** SUN proteins (magenta) combined with DAPI (gray). A continuous signal around the entire NE is present in both normal-looking and hypercondensed *sar1-4* meiocytes, as well as in the WT. **(B)** NPCs (green) combined with DAPI (gray). Normal-looking *sar1-4* meiocytes are indistinguishable from WT cells, whereas in hypercondensed *sar1-4* meiocytes NPCs appear to be absent. Scale bars = 5 µm.

Since the absence of the outer ring complex nucleoporins may compromise the integrity of the NPCs, we decided to analyze the distribution of these complexes, as well as that of the LINC complexes in the NE of *sar1* meiocytes. For the study of LINC complexes, we applied an immunolocalization to detect SUN proteins (Figure 6; Supplementary Figure S7). In WT meiocytes these proteins present a distribution pattern along the entire NE during prophase I, disappearing at the end of this stage (n = 107). In the case of *sar1*, no differences were found in the pattern of these proteins with respect to WT, with a continuous signal also appearing around the entire NE, both in normal-looking meiocytes (n = 25) and in hypercondensed meiocytes (n = 9). As expected, we observed a reduction of the NE surface in the latter, in line with their hypercondensed chromatin state. Thus, the distribution of LINC complexes is apparently not affected by the absence of a structural nucleoporin.

Regarding NPCs, in WT cells the distribution pattern is similar to that of the LINC complexes, with a signal appearing along the entire NE during prophase I (Figure 6). We confirmed that normal-looking *sar1* meiocytes (n = 103) do not display variations in the distribution pattern of NPCs with respect to WT meiocytes (n = 83). However, in *sar1* hypercondensed meiocytes, no trace of the signal corresponding to NPCs was detected at any location in the nucleus (n = 76), revealing that these cells present severe structural abnormalities in the NPCs.

### The interplay between SAR1 and AXR1

Nucleoporins SAR1 and SAR3 are called by these names because they were firstly identified in a screening for suppression of the *axr1* resistance to auxin phenotype (Cernac et al., 1997; Parry et al., 2006). The *axr1* mutation produces a dramatic effect on plant morphology (Lincoln et al., 1990) and, interestingly, meiotic defects consisting of abnormal synapsis at prophase I, univalents at metaphase I, unequal chromosome segregation at anaphase I, and unbalanced tetrads or polyads (Jahns et al., 2014). The origin of these meiotic abnormalities is poorly understood, although it has been suggested that they could be related to the protein modifications associated to the RUBylation pathway (Jahns et al., 2014).

**FIGURE 7:**
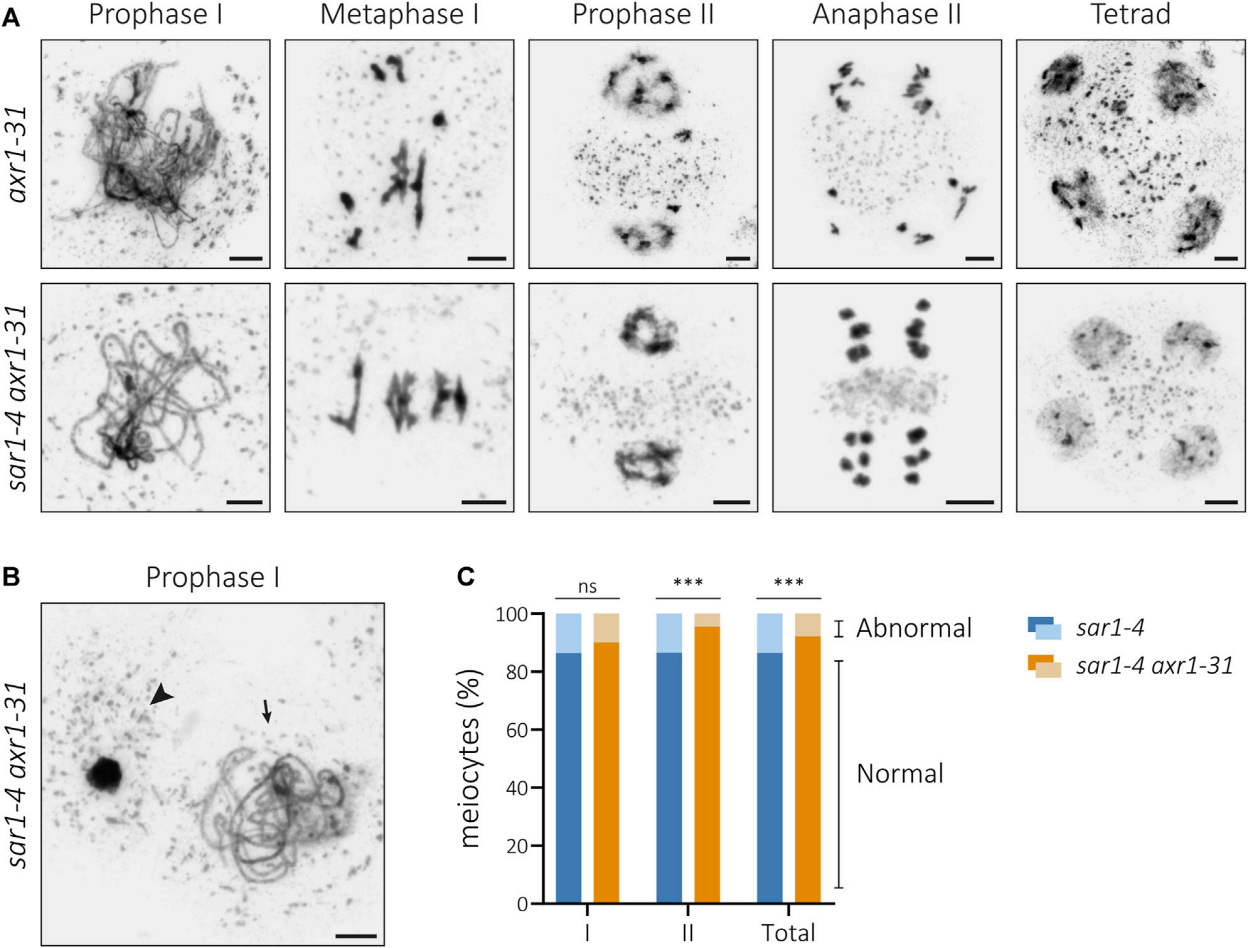
Cytological analysis of PMCs from the double mutant *sar1-4 axr1-31*. **(A)** Chromosome spreads of male meiocytes in *axr1-31* and *sar1-4 axr1-31*. The single mutant *axr1-31* shows defects in synapsis, presence of univalents at metaphase I, unbalanced nuclei during the second meiotic division, and tetrads with micronuclei. These meiotic defects are suppressed in the double mutant *sar1-4 axr1-31*. **(B)** Example of hypercondensed (arrowhead) and normal-looking (arrow) meiocyte in the double mutant *sar1-4 axr1-31*. **(C)** Proportion of abnormal and normal meiocytes in *sar1-4* (blue) and *sar1-4 axr1-31* (orange). Fisher’s exact test was performed to analyze differences between the mutants (*p*-value: ns–non-significant, ***p < 0.001). I: Meiosis I; II: Meiosis II. Scale bars = 5 µm.

Since mutations in either *SAR1* or *SAR3* suppress most aspects of the phenotype conferred by *axr1* (Cernac et al., 1997; Parry et al., 2006; Supplementary Figure S8), we wondered if this suppression also affects *axr1* meiotic defects. To find out if this was the case, we generated the double mutant *sar1 axr1* and analyzed its meiotic phenotype. The results showed that the characteristic meiotic problems associated with *axr1* disappear in *sar1 axr1* (Figure 7A). In the double mutant, we observed full synapsis at pachynema, five bivalents at metaphase I, equal distribution of chromosomes during both meiotic divisions, and balanced tetrads. This means that in the double mutant the asynaptic phenotype of *axr1* disappears, as well as the problems in bivalent formation.

To analyze the different chromosome configurations in more detail, the FISH technique was applied (Supplementary Figure S8). In WT meiocytes most of the bivalents are closed (ring bivalents), with at least one chiasma in each arm (76.2%; n = 69), and the same occurs in *sar1* (85.5%; n = 43), in which no univalents are detected either. In *axr1* most of the bivalents are open (rod bivalents), without chiasmata in one of the arms (47.2%; n = 72), although there are also closed bivalents (24.2%) and univalents (28.6%). In the double mutant *sar1 axr1,* most bivalents appeared in closed configuration (70.4%; n = 50), recovering the WT phenotype (*p* = 0.155). Obligatory chiasma formation is almost restored in this double mutant, as univalents disappear. We detected only 4% of the cells with a single pair of univalents (2/50). To further analyze recombination events, we conducted an immunolocalization to detect MLH1, a marker of most crossovers in Arabidopsis (Jackson et al., 2006). This analysis showed no significant differences between the WT (8.09 ± 0.40), *sar1* (8.03 ± 0.38), and *sar1 axr1* (8.42 ± 0.25) (F = 0.469, *p* = 0.627) concerning MLH1 foci (Supplementary Figure S8).

Regarding to the presence of abnormal meiocytes, in the double mutant *sar1 axr1* there was a decrease in the percentage of these meiocytes, as well as in *sar1 spo11* (Figures 7B, C). We observed fewer abnormal meiocytes than in the *sar1* single mutant in the first meiotic division, although the difference between both mutants was not significant. The reduction was statistically significant in the second meiotic division (Supplementary Table S8). Furthermore, in *sar1 axr1*, the proportion of hypercondensed meiocytes was higher than that of fragmented meiocytes in the second division, in contrast to the *sar1* single mutant (Supplementary Table S5).

## Discussion

The results of this work have revealed that some of the structural nucleoporins that belong to the outer ring complex of the NPC are necessary for proper meiosis progression. Several of the nucleoporin-defective mutants of this complex show a pleiotropic phenotype, including developmental deficiencies and reduced fertility (Cernac et al., 1997; Parry et al., 2006). We have demonstrated that the semi-sterile phenotype is due to failures in meiosis, highlighting the importance of the NPCs in this cell division.

### SAR1 and SAR3, as well as HOS1, are necessary to ensure the proper progression of meiosis

The cytological analysis of PMCs in *sar* mutants has determined the presence of abnormal meiocytes, both in first and second division. The altered meiotic phenotype is characterized by the presence of cells with extreme chromatin condensation, especially during the first division, in addition to the appearance of chromosomal fragments from anaphase I onwards, which generates unbalanced tetrads and even polyads at the end of meiosis. These abnormal meiocytes appear along with others in which meiosis is properly achieved. The percentage of meiocytes with alterations varies between the mutants *sar1* and *sar3* and increases considerably in the double mutant *sar1 sar3* (Figure 1), which might suggest a certain degree of independence in their functions. In the double mutant, problems in vegetative development and fertility are also exacerbated respect to the single mutants, suggesting that the loss of both nucleoporins produce a severe defect in NPC function (Parry et al., 2006). We also found similar meiotic alterations in *hos1* (Supplementary Figure S2). HOS1 is an outer ring complex nucleoporin that functions as an E3 ubiquitin ligase preventing precocious flowering. Interestingly, SAR1 and SAR3 also contribute to flowering time regulation by ensuring the stability and association of HOS1 with the NPC (Cheng et al., 2020; Li et al., 2020). It is possible that these nucleoporins affect a common regulatory mechanism between flowering and meiosis, since we have not detected meiotic problems in other mutants defective in the outer ring complex (*nup85*, *seh1*), which, unlike the previous ones, do not exhibit a significant flowering phenotype (Li and Gu, 2020). These mutations also do not aggravate the somatic abnormalities observed in *sar1*, suggesting some functional diversity between these nucleoporins that belong to the same NPC subcomplex (Wiermer et al., 2012; Parry, 2014). On the other hand, an accumulation of polyadenylated mRNA was found in all outer ring complex mutants in which RNA export was analyzed (Parry, 2015). Therefore, the observed meiotic alterations do not seem to be related to this mRNA accumulation (Supplementary Figure S3). It is likely that these nucleoporins do not only function in the context of NPCs. Indeed, NPCs undergo large-scale structural rearrangements during cell division and, for example, the nuclear basket transiently dissociate from the NPC core during meiosis in budding yeast (King et al., 2023). It is not known whether something similar occurs in Arabidopsis, but in any case, HOS1, apart from its E3-ubiquitin ligase activity, is associated with chromatin to influence gene expression in this species (Lazaro et al., 2012; Jung et al., 2014). In the case of SAR1 and SAR3, no such association has been confirmed. Further experiments will be required to confirm whether the meiotic functions of these nucleoporins are related to their structural function within the NPC.

*sar1* and *sar3* present abnormalities in mRNA accumulation or nuclear morphology in all somatic cells (Cernac et al., 1997; Parry et al., 2006; Parry, 2014). However, they only show meiotic alterations in a percentage of meiocytes. Although there is no clear explanation for this result, it is possible that in some meiocytes other nucleoporins cannot supply the function of SAR proteins. Alternatively, it could be a timing problem, with defects occurring in meiocytes in which meiosis is slower. In any case, the presence of normal-looking meiocytes together with abnormal meiocytes has been described in other mutants. For example, mutations in *BQT1,* a gene encoding a protein that tether telomeres to the spindle-pole body during prophase I, affect spindle formation in about half of meiotic cells in fission yeasts (Klutstein et al., 2015). In Arabidopsis, mutants lacking JASON, a protein essential for proper spindle orientation, or NSE2, a protein belonging to the SMC5/6 complex, generate normal and unreduced meiotic products (Erilova et al., 2009; De Storme and Geelen, 2011; Yang et al., 2021). Mutants affected in *CYCA2* genes or *CYCB3;1* also show alterations in a fraction of meiocytes (Bulankova et al., 2013).

The suppression of chromosome fragmentation in *sar1 spo11* has evidenced that fragments produced by the absence of the nucleoporin are generated by the inability to properly repair the recombination intermediates formed from meiotic DSBs (Figure 4). These defects in DNA repair may be due to failures in the recruitment of proteins involved in the early stages of HR, as evidenced by a reduction in the number of γH2AX and RAD51 foci (Figure 5). Another possibility is that, due to the absence of the nucleoporin, HR proceeds more slowly in some meiocytes and this triggers failures in the repair of DSBs. In any case, despite these problems in HR, the chromosome axes seem to form correctly, even in the abnormal meiocytes, according to the results obtained for ASY1. However, the process of synapsis is compromised (Figure 2). The absence of synapsis is most likely caused by problems in DNA homology search during DSB repair, which is a prerequisite for the progression of synapsis (Osman et al., 2011). In addition, the entangled chromosomes observed at metaphase I in *sar1*, *sar3*, and *hos1* (Figures 1, 3; Supplementary Figure S2) are reminiscent of those observed in recombination-defective mutants such as *rad51*, *xrcc3*, *rad51c* or *mnd1* (Kerzendorfer et al., 2006; Pradillo et al., 2012; 2014). Interestingly, SUMOylation in plants, as well as in yeast, appears to be linked to the NPC, and SUMOylated proteins accumulate in mutants defective for NUA (structural component of the nuclear basket) and SAR1 (Muthuswamy and Meier, 2011). In fission yeast, the Y-complex nucleoporin Nup132 is involved in the regulation of SUMOylation during meiosis, and mutants deficient for this nucleoporin exhibit upregulated SUMOylated proteins including Pim1, Top1, and Top2 (Yang et al., 2023). Hyper-SUMOylation of Top2 alters meiotic chromosome architecture (Li et al., 2013). In Arabidopsis, *topII* mutants show condensation defects, entangled chromosomes, and high levels of DNA fragmentation (Martinez-Garcia et al., 2018). It is tempting to speculate that the meiotic phenotype observed in Arabidopsis Y-complex deficient mutants is somehow related to alterations in the SUMOylation of proteins with meiotic function.

### Distribution of NPCs is altered in hypercondensed meiocytes

The excessive chromatin condensation observed in *sar1* does not seem to originate from the problems in HR, as it does not disappear in the *sar1 spo11* double mutant (Figure 4). These abnormally condensed meiocytes have a morphology similar to that of cells undergoing cell death. This process is characterized by cell shrinkage, chromatin condensation, and DNA fragmentation (Kerr et al.,1972; Reape et al., 2008). Hendzel et al. (1998) pointed out that during cell death, chromatin condensation is not an active process associated with histone phosphorylation as occurs in mitosis or meiosis (Houben et al., 1999; Manzanero et al., 2000; Oliver et al., 2013). In this case, condensation would be the result of the degradation of euchromatin, nuclear matrix and lamin, in addition to the aggregation of heterochromatin. However, we have not detected appreciable variations in euchromatin- or heterochromatin-specific epigenetic marks in the abnormally condensed cells, at least at the cytological level, as variations at the molecular level cannot be ruled out (Supplementary Figure S5). Chromosome condensation problems in meiosis have been described in mutants defective for the condensin complex (Siddiqui et al., 2003; Smith et al., 2014) or *mmd1* (*male meiocyte death1*) mutants (Yang et al., 2003; Wang et al., 2016). Nevertheless, unlike *sar* mutants, these mutants do not show any defects in the appearance of chromosomes during early prophase I and present chromatin decondensation at later stages (Yang et al., 2003; Smith et al., 2014).

The altered distribution of NPCs in the NE could be the source of the problems in chromatin compaction, since in abnormally condensed meiocytes there is no defined pattern for NPCs, unlike in normal-looking meiocytes (Figure 6). This phenotype reveals the importance of SAR1 in the structure of NPCs. The absence of signal corresponding to NPCs in these meiocytes is not due to NE disintegration, as the signal corresponding to SUN proteins is observed around the chromatin. Perhaps the apparent collapse of NPCs could have some reversibility, and this explains why meiotic changes only occur in a percentage of cells. Indeed, in HeLa cells depletion of the Nup107-160 complex results in nuclei with a continuous NE but no NPCs, although the defect in NPC assembly could be reversed by adding Nup107-160 complex containing fractions (Walther et al., 2003). In addition, depletion of this complex also induces cell death following a spindle checkpoint-dependent delay during mitosis (Rieder and Maiato, 2004; Zuccolo et al., 2007). In plants, the spindle checkpoint is not as tightly regulated as in yeast or animals, and it could even not function or be much relaxed during meiosis (Komaki and Schnittger, 2016). In this sense, mutants with severe recombination problems can complete meiosis, although no gamete is functional (Wijnker and Schnittger, 2013). In *sar* mutants the hypercondensed meiocytes appear to progress through meiosis, giving rise to the masses of entangled chromosomes observed at metaphase I (Figure 1). This is of particular interest because studying meiosis in these mutants may provide information on the possible meiotic function of these nucleoporins that cannot be obtained from studies using other model organisms.

### SAR1, AXR1, and the auxin response

AXR1 is a component of the RUBylation pathway targeting, among others, cullin proteins (Mergner and Schwechheimer, 2014). *axr1* plants display auxin-related growth defects that are suppressed by eliminating the function of SAR1 or SAR3 (Cernac et al., 1997; Parry et al., 2006). In the present work, we have shown that the *sar1* mutation also reverses the altered meiotic phenotype of *axr1*, which is completely different from that of *sar1* (Jahns et al., 2014). In *sar1 axr1* the synapsis problems disappear and the formation of the obligatory chiasma, required for bivalent formation, is restored (Figure 7). In the case of the somatic phenotype, it has been suggested that sar mutants delay the nuclear import of Aux/IAA negative regulators, thus ameliorating the defect in *axr1*, which initially inhibits auxin gene expression (Parry et al., 2006). The reversal of the *axr1* meiotic phenotype in *sar1 axr1* may also be explained in this way. On the other hand, neither *sar1* nor *sar3* exhibit auxin hypersensitivity, revealing a complex relationship between the NPC and the auxin response (Parry et al., 2006; Robles et al., 2012). Surprisingly, there are no studies showing a clear link between auxins and meiosis. This deserves further investigation in the future.

Alternatively, the reversal of the meiotic phenotype in *sar1 axr1* might be related to the RUBylation pathway. It has been suggested that AXR1 functions during meiotic recombination through the activation of a CRL4 (CULLIN RING LIGASE4) complex involved in the ubiquitylation of specific protein targets, since a *cul4* mutant exhibit a meiotic phenotype reminiscent of that observed in *axr1* (Jahns et al., 2014). Curiously, the SUMO and ubiquitin-proteasome systems function coordinately in meiotic chromosome organization and the regulation of meiotic recombination in mouse (Prasada Rao et al., 2017). The presence of upregulated SUMOylated proteins in *sar1* may somehow compensate for the lack of CRL4 activity in *axr1*. Further analyses will be required to connect the function of these post-translational modifications to the NPCs.

## Concluding remarks

During meiosis, LINC complexes contribute to promote telomere-driven chromosome movement at prophase I and this function is highly conserved in evolution (Kim et al., 2022). Strikingly, these complexes are also required for the distribution of NPCs in the NE (Liu et al., 2007). Surprisingly, few studies have analyzed the distribution of NPCs in plants, although they appear to have a non-homogeneous distribution during the early stages of meiosis (Holm, 1977; Zickler and Kleckner, 1998; Cowan et al., 2002). Similarly, little is known about how NPCs can influence chromosome behavior during meiosis. This study reveals a meiotic role for SAR1 and SAR3, scaffold nucleoporins belonging to the outer ring complex, in plant meiosis. These findings will lead to new lines of research to better understand how NE organization is modulated in the dynamic chromosome events during meiosis and the specific function of NPCs in this type of cell division.

## Data Availability

The original contributions presented in the study are included in the article/Supplementary Material, further inquiries can be directed to the corresponding author.
