# Supplementary material for: The scaffold nucleoporins SAR1 and SAR3 are essential for proper meiotic progression in Arabidopsis thaliana
Source: Front Cell Dev Biol. 2023 Dec 4;11:1285695. doi: 10.3389/fcell.2023.1285695 (PMC10725928; doi:10.3389/fcell.2023.1285695)
Supplement: Supplementary file 1 [file DataSheet1.pdf]

Supplementary Material

The scaffold nucleoporins SAR1 and SAR3 are essential for proper meiotic progression in Arabidopsis *thaliana*

Nadia Fernández-Jiménez[1], Marina Martinez-Garcia[2], Javier Varas[3], Félix Gil-Dones[1], Juan Luis Santos[1], Mónica Pradillo[1*]

[1]Department of Genetics, Physiology and Microbiology, Faculty of Biological Sciences, Universidad Complutense de Madrid, Spain

[2]Department of Biotechnology-Plant Biology, School of Agricultural, Food and Biosystems Engineering, Universidad Politécnica de Madrid, Spain

[3]GlaxoSmithKline Spain, Tres Cantos, Madrid, Spain

*** Correspondence:**
pradillo@bio.ucm.es

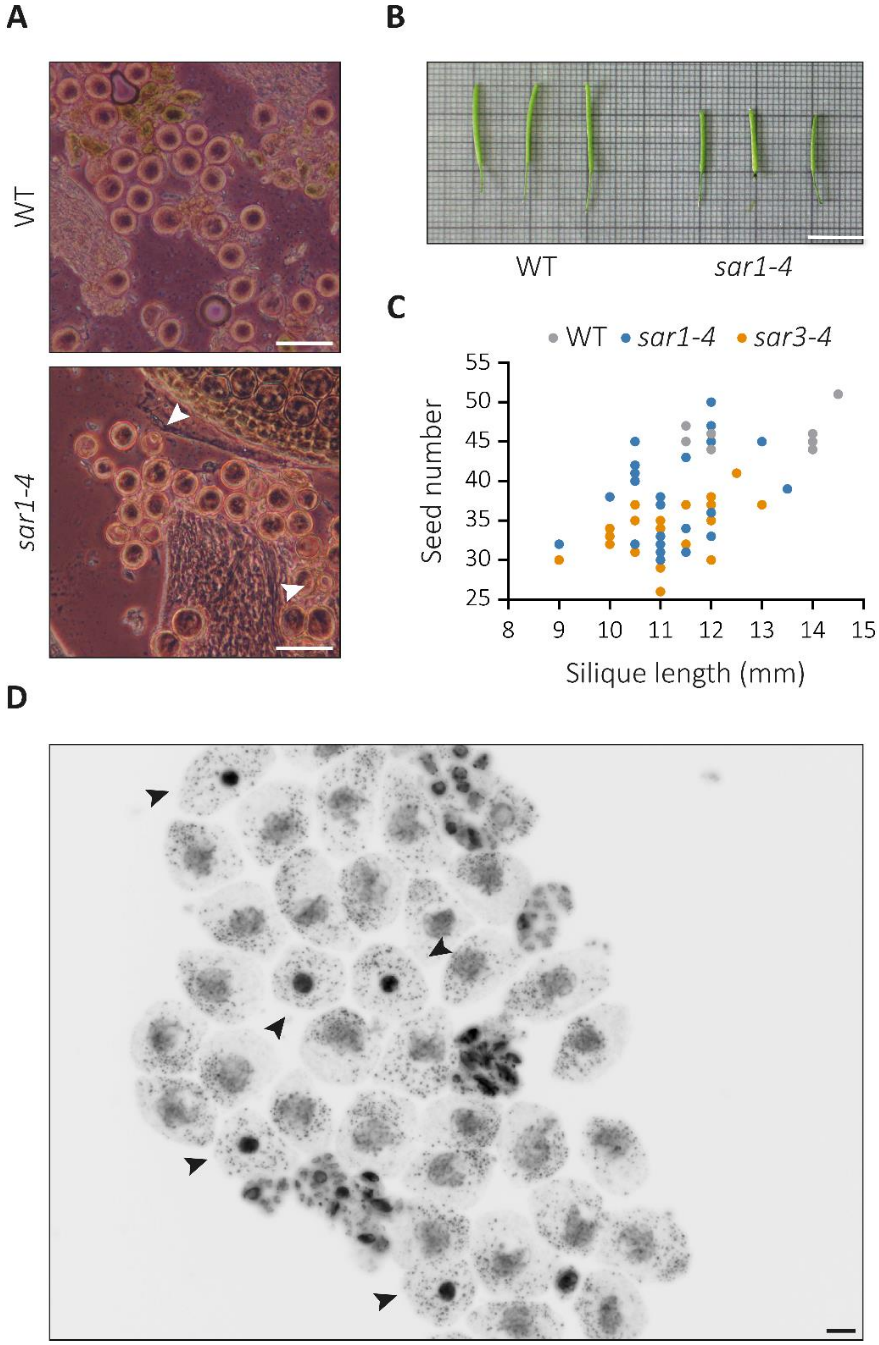

Supplementary Figure S1. Defects in fertility and meiosis in *sar* mutants. **(A)** Aceto-carmine staining of anthers to analyze pollen grains. *sar1-4* shows pollen grains smaller than those observed in the WT (arrowheads). Scale bars = 50 µm. **(B)** Siliques from WT and *sar1-4*. Scale bar = 1 cm. **(C)** Evaluation of fertility considering silique length (mm) and seed number per silique. Three different plants of each genotype were analyzed. The mutants have lower values than the WT in both parameters. **(D)** Prophase I *sar1-4* meiocytes from the same pollen sac obtained after applying squash technique and DAPI staining. Normal-looking and abnormal meiocytes (arrowheads) are observed. Scale bar = 5 µm.

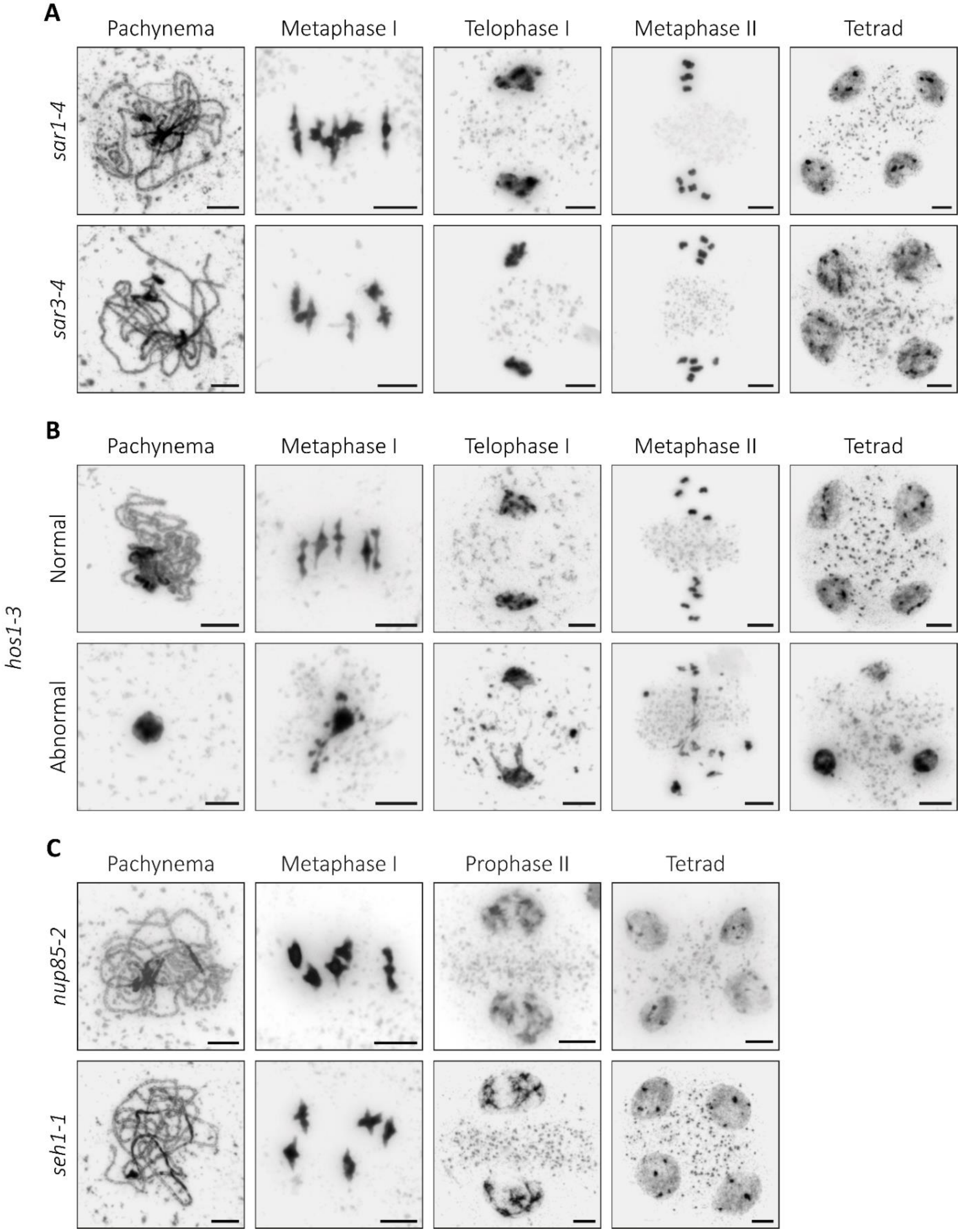

Supplementary Figure S2. Cytological analysis of PMCs from mutants defective in nucleoporins belonging to the outer ring complex. **(A)** Normal-looking meiocytes from *sar1-4* and *sar3-4*. **(B)** Normal-looking (first row) and abnormal (second row) meiocytes in *hos1-3*, showing hypercondensation and chromosome fragmentation. **(C)** Normal meiosis in *nup85-2* and *seh1-1*. Scale bars = 5 µm.

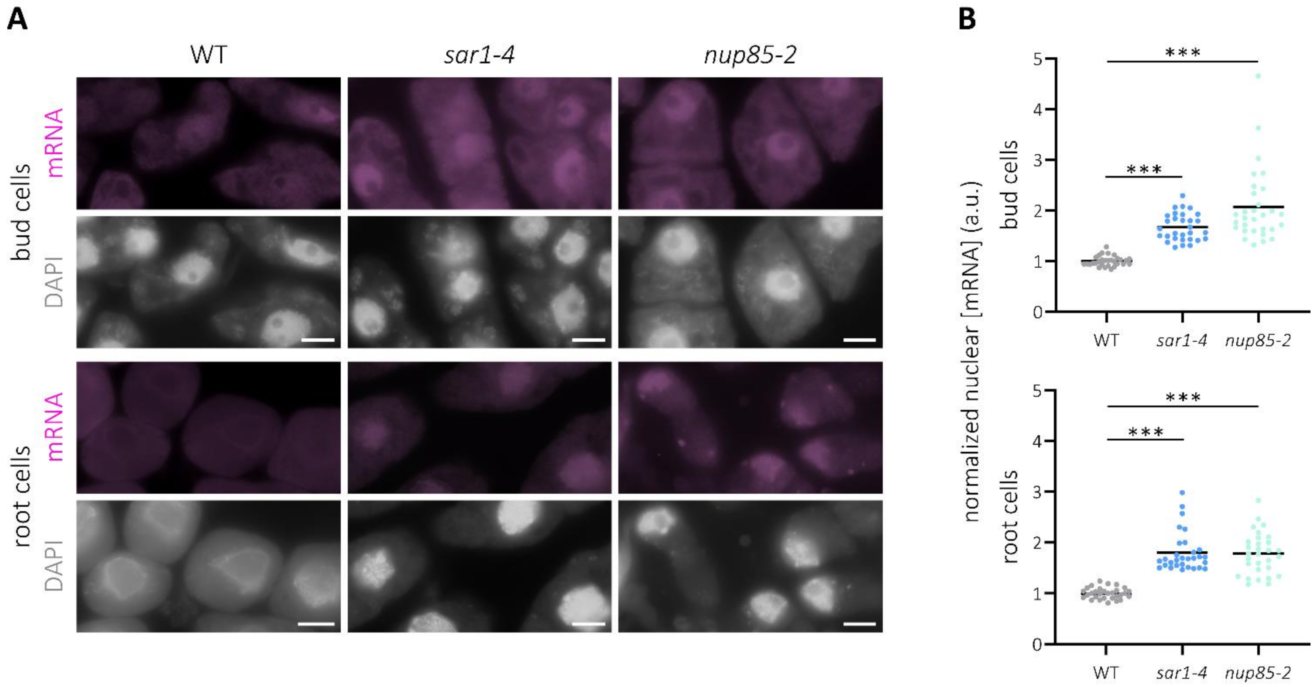

Supplementary Figure S3. Analysis of mRNA accumulation in nuclei from *sar1-4* and *nup85-2*. **(A)** mRNA labelling with an oligo(dT) probe (magenta) combined with DAPI (gray) in flower bud and root cells. **(B)** Quantification of mRNA in *sar1-4* and *nup85-2* nuclei, normalizing with WT nuclei (a.u.: arbitrary units). Both mutants show a significant mRNA accumulation inside the nucleus. Scale bars = 5 µm.

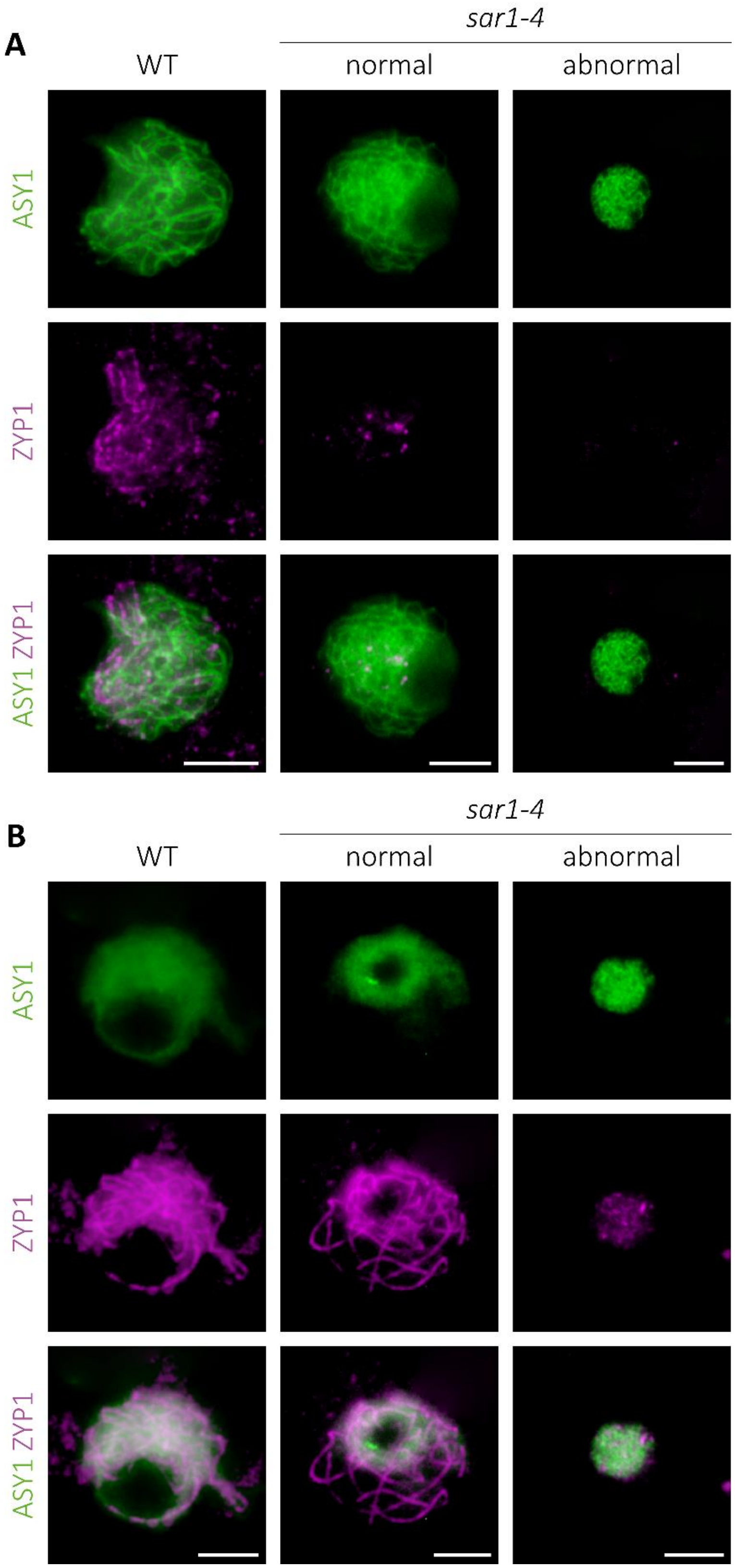

Supplementary Figure S4. Individual channels related to immunolocalization of meiotic chromosome axes and synaptonemal complex. Merged pictures are displayed in Figure 2. **(A)** Zygonema. **(B)** Pachynema. Scale bars = 5 µm.

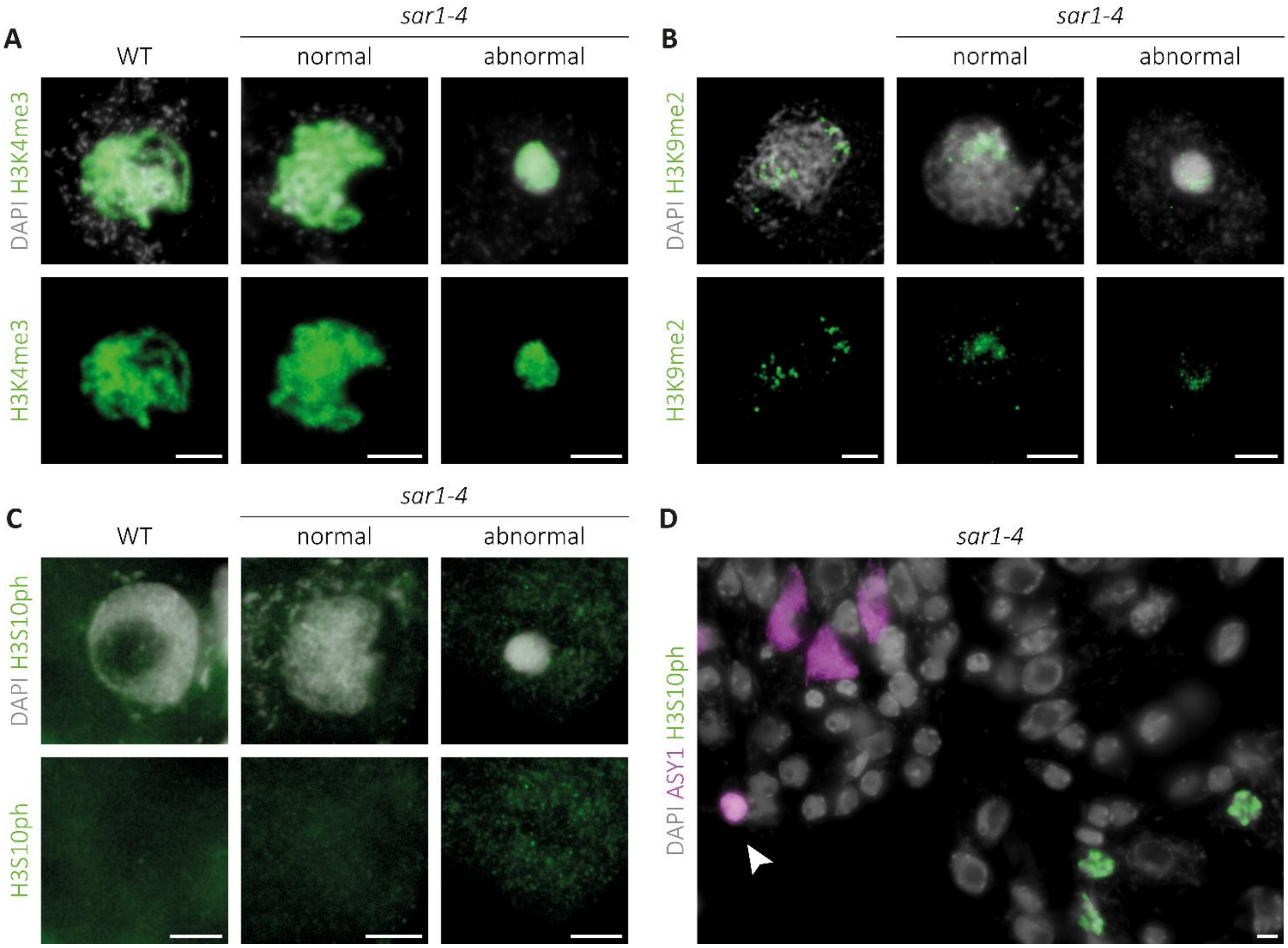

Supplementary Figure S5. Immunolocalization of histone modifications. Squash preparations of WT, normal-looking and hypercondensed *sar1-4* zygotene cells showing epigenetic marks characteristic of euchromatin, heterochromatin, and chromatin condensation. Hypercondensed *sar1-4* meiocytes show a normal pattern with respect to these epigenetic marks. **(A)** Euchromatin mark (H3K4me3, green). **(B)** Heterochromatin mark (H3K9me2, green). **(C)** Chromatin condensation mark (H3S10ph, green). **(D)** *sar1-4* flower bud cells, including normal-looking and abnormal (arrowhead) zygotene cells detected with ASY1 (magenta) and cells in diakinesis in which H3S10ph signal (green) is observed. Scale bars = 5 µm.

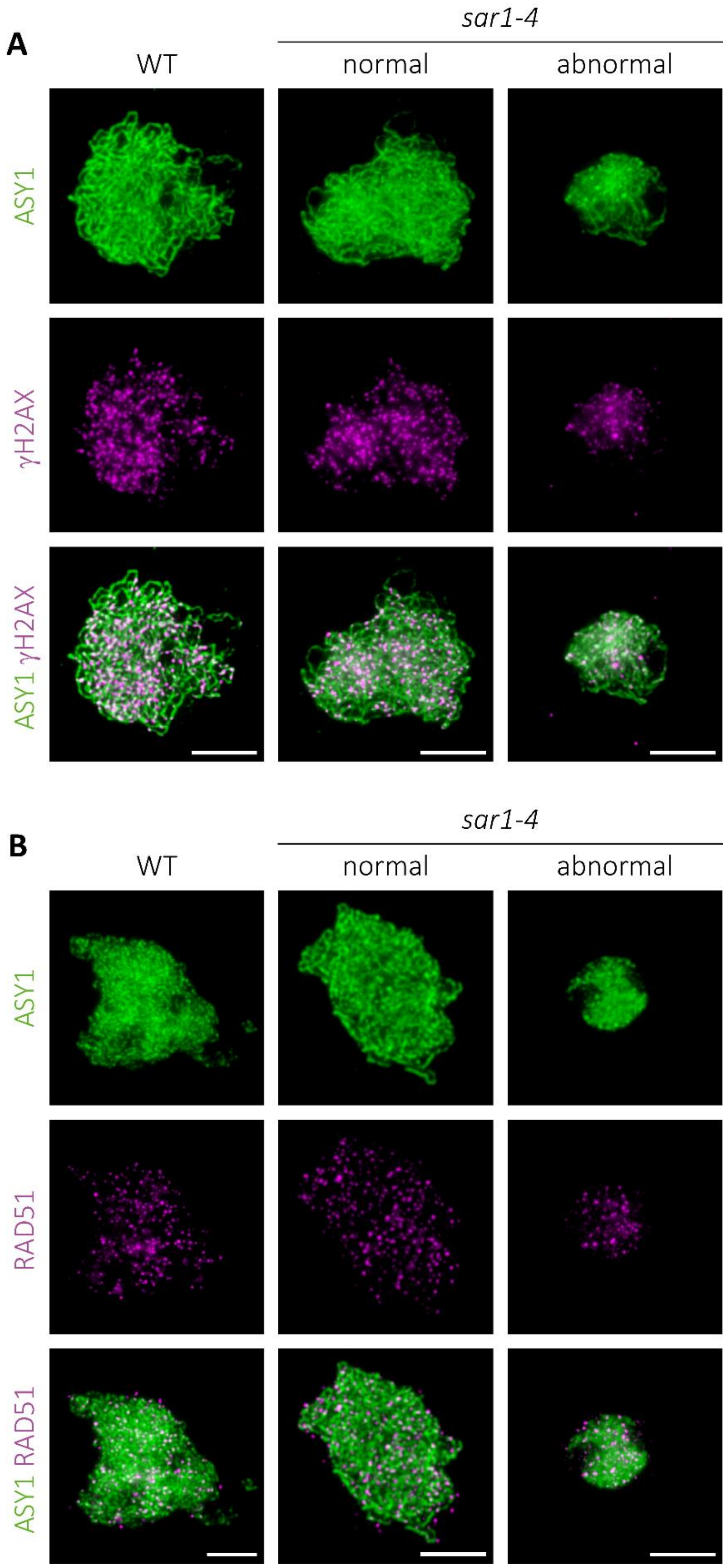

Supplementary Figure S6. Individual channels related to immunolocalization of γH2AX and RAD51. Merged pictures are displayed in Figure 5. **(A)** Immunolocalization of ASY1 and γH2AX. **(B)** Immunolocalization of ASY1 and RAD51. Scale bars = 5 µm.

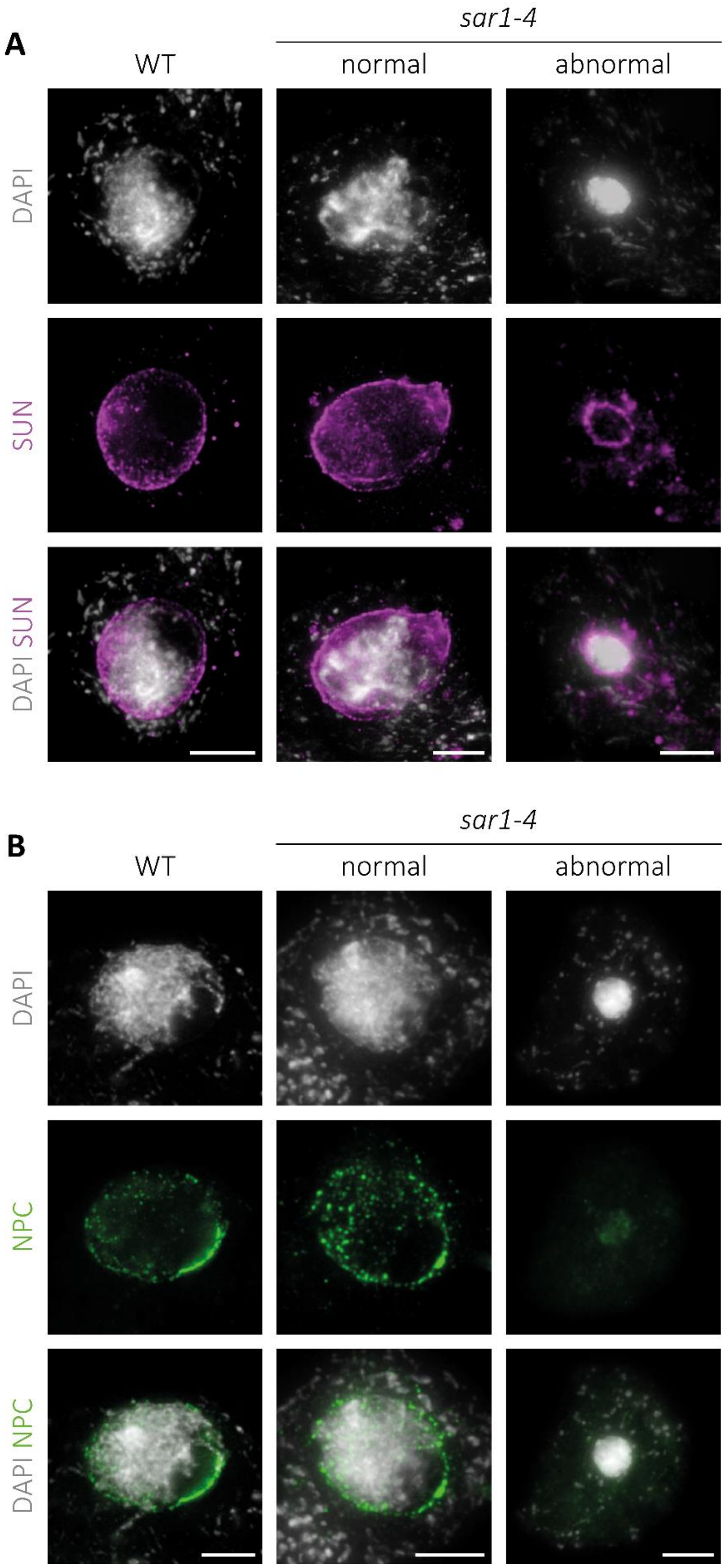

Supplementary Figure S7. Individual channels related to immunolocalization of SUN proteins and NPCs. Merged pictures are displayed in Figure 6. **(A)** Immunolocalization of SUN proteins. **(B)** Immunolocalization of NPCs. Scale bars = 5 µm.

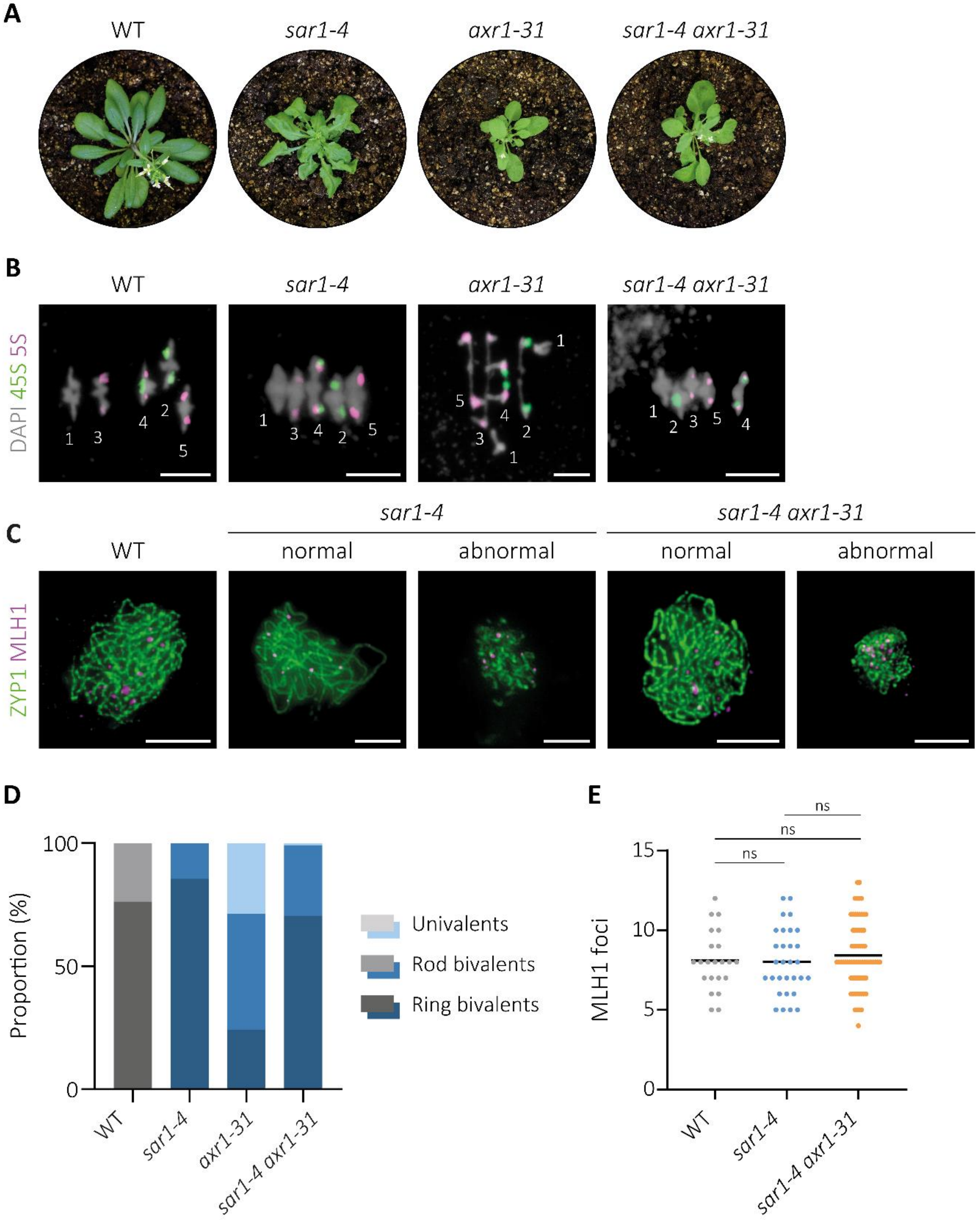

Supplementary Figure S8. Representative pictures of the rosette appearance and cytogenetic analyses in *sar1-4*, *axr1-31*, and *sar1-4 axr1-31*. **(A)** Rosettes from WT, *sar1-4*, *axr1-31*, and *sar1-4 axr1-31*. **(B)** WT, *sar1-4*, *axr1-31*, and *sar1-4 axr1-31* meiocytes at metaphase I. Numbers identify each bivalent. **(C)** Immunolocalization of MLH1 (magenta) and ZYP1 (green) in chromosome spreads from WT, *sar1-4*, and *sar1-4 axr1-31*. **(D)** Chromosome configurations in WT, *sar1-4*, *axr1-31*, and *sar1-4 axr1-31* meiocytes. It is noteworthy the presence of univalents in *axr1-31*. These univalents disappear in the double mutant, which has chromosome configurations similar to those of WT (see the text for more details). **(E)** Quantification of MLH1 foci (*p* value: ns – non-significant). Scale bars = 5 µm.

Supplementary Table S1. Primers used for genotyping. In addition, the primer LBb1.3 (5´-ATTTTGCCGATTTCGGAAC-3´) was used as a T-DNA border primer. RP: right primer; LP: left primer.

Allele	Sequence 5'-3'	T-DNA line
axr1-31	RP - TCATGTGGAGAATGGGCTTAC<br>LP - TGTGATTGAATATTGCAGGAGC	SALK_013238
hos1-3	RP - AAGGCACGATAATGGTCTTCC<br>LP - TGGACCGCATATTTCAGGTAG	SALK_069312
nup85-2	RP - ATTCGTGGCATTTTTGAAATG<br>LP - TGTATCAGACTGCGTGGTGAG	SALK_133369
sar1-4	RP - CAGCCCTTGAGCAAGTAGATG<br>LP - TTACCTGAATATGTCCACCCG	SALK_126801
sar3-4	RP - ACCTCCCGACAGTTCATTACC<br>LP - TGTGGAAGCCAAGGAGTTATG	SALK_117966
seh1-1	RP - TTGAGCTCTTGAATCCATTGG<br>LP - ATCTGTGATTGCAAACGATCC	SALK_022717
spo11-1-5	RP - CAGTTTCTCTCAGGCATTTCG<br>LP - AAATGCCACAATGGAGGTATG	SALK_009440

Supplementary Table S2. Primary antibodies used in this study.

Antibody	Animal	Working Dilution	Source
α-NPC	Mouse	1:100	Santa Cruz Biotechnology
α-SUN	Rabbit	1:300	Agrisera
α-H3K4me3	Rabbit	1:300	
α-H3K9me2	Rabbit	1:300	
α-H3S10ph	Rabbit	1:300	
α-γH2AX	Mouse	1:500	Merck
α-RAD51	Rabbit	1:300	Kindly donated by Prof. Chris Franklin (University of Birmingham, UK)
α-MLH1	Rabbit	1:300	
α-ASY1	Rabbit /Rat	1:500	
α-ZYP1	Rabbit /Rat	1:500	

Supplementary Table S3. Proportion of abnormal meiocytes in *sar1-4* and *sar3-4.*

	sar1-4			***sar3-4***			*p*
	normal	abnormal	n	normal	abnormal	n	
I	86.42%	13.58%	346	78.25%	21.75%	1,007	0.001
II	86.55%	13.45%	290	88.25%	11.75%	366	0.553
Total	86.48%	13.52%	636	80.92%	19.08%	1,373	0.002

I: first meiotic division; II: second meiotic division; n: cell number analyzed; *p*: P value from a Fisher's exact test.

Supplementary Table S4. Proportion of abnormal meiocytes in *sar1-4 sar3-4.*

	sar1-4 sar3-4			*p*	
	normal	abnormal	n	*sar1-4*	*sar3-4*
I	46.67%	53.33%	90	< 0.0001	< 0.0001
II	29.17%	70.83%	24	< 0.0001	< 0.0001
Total	42.98%	57.02%	114	< 0.0001	< 0.0001

I: first meiotic division; II: second meiotic division; n: cell number analyzed; *p*: P value from a Fisher's exact test.

Supplementary Table S5. Abnormalities (hypercondensation, chromosomal fragmentation, and hypercondensation + chromosomal fragmentation) in meiocytes from *sar1-4*, *sar3-4*, *sar1-4 sar3-4*, and *sar1-4 axr1-31*.

		sar1-4	*sar3-4*	*sar1-4 sar3-4*	*sar1-4 axr1-31*
I	hypercondensed	93,62%	99,09%	60,42%	98,40%
	fragmented	0,00%	0,00%	31,25%	0,80%
	h + f	6,38%	0,91%	8,33%	0,80%
II	hypercondensed	27,59%	21,74%	35,29%	43,48%
	fragmented	34,48%	30,43%	47,06%	34,78%
	h + f	37,93%	47,83%	17,65%	21,74%

I: first meiotic division; II: second meiotic division; h: hypercondensed; f: fragmented

Supplementary Table S6. Proportion of abnormal meiocytes in *sar1-4* and *sar1-4 spo11-1-5*.

	sar1-4			***sar1-4 spo11-1-5***			*p*
	normal	abnormal	n	normal	abnormal	n	
I	86.42%	13.58%	346	86.80%	13.20%	765	0.849
II	86.55%	13.45%	290	95.50%	4.50%	222	0.001
Total	86.48%	13.52%	636	88.13%	11.87%	994	0.357

I: first meiotic division; II: second meiotic division; n: cell number analyzed; *p*: P value from a Fisher's exact test.

Supplementary Table S7. Statistical analysis of γH2AX and RAD51 foci in *sar1-4*.

**γH2AX**			
ANOVA	F	*p*	
	59.95	<0.0001	
Tukey's post-hoc test	WT	*sar1-4* normal	*sar1-4* abnormal
WT			
sar1-4 normal	0.9232		
sar1-4 abnormal	<0.0001	<0.0001	
**RAD51**			
ANOVA	F	*p*	
	18.05	<0.0001	
Tukey's post-hoc test	WT	*sar1-4* normal	*sar1-4* abnormal
WT			
sar1-4 normal	0.958		
sar1-4 abnormal	<0.0001	<0.0001	

Supplementary Table S8. Proportion of abnormal meiocytes in *sar1-4* and *sar1-4 axr1-31*.

	sar1-4			*sar1-4 axr1-31*			*p*
	normal	abnormal	n	normal	abnormal	n	
I	86.42%	13.58%	346	90.02%	9.98%	1,252	0.0624
II	86.55%	13.45%	290	95.55%	4.45%	786	<0.0001
Total	86.48%	13.52%	636	92.15%	7.85%	2,038	<0.0001

I: first meiotic division; II: second meiotic division; n: cell number analyzed;
p: P value from a Fisher's exact test.
